# Investigating the functional neural network dynamics of cognitive-affective action planning during threat processing

**DOI:** 10.3389/fpsyg.2026.1782939

**Published:** 2026-03-27

**Authors:** John Foley, Siraj J. Lyons, Olivia Cook, Brendan E. Depue

**Affiliations:** 1 Department of Psychological & Brain Sciences, University of Louisville, Louisville, KY, United States; 2 Surgical Theater, Beachwood, OH, United States; 3 Department of Anatomical Sciences & Neurobiology, University of Louisville, Louisville, KY, United States

**Keywords:** emotion, fMRI, functional connectivity, neural network, threat

## Abstract

**Introduction:**

The current investigation used functional neuroimaging data from four different tasks involving threatening/fearful faces and screams to understand the functional organization of neural networks.

**Methods:**

Emphasis was placed on graph theory metrics of both local connectivity (participation coefficient, betweenness centrality) and global connectivity (global efficiency, modularity) to assess intra- and inter-network connectivity across a highly parcellated (412 nodes) brain template. A total of 124 participants (57% female; *M*_Age_ = 22.06) were included in the investigation. Both mass univariate linear mixed effects models and multivariate classification via support vector machine were employed to uncover the nature of network dynamics across 8 well described intrinsic connectivity networks (ICNs).

**Results:**

Our findings suggest that a diffuse pattern of strengthened intra-network connectivity defined social threat processing. Univariate analyses exhibited a preponderance of nodes within the somatomotor network, indicating the potential for motor preparation during social threat processing. Multivariate classification exhibited more diffuse focal participation from limbic, default, visual, attentional, control and somatomotor ICNs.

**Conclusion:**

Our results suggest the role of intra- and inter-network functional organization in response to threat and carries implications for viewing various networks through dynamic perspectives.

## 1 Introduction

Due to the richness of human social interaction, faces and vocal prosody are exceptionally salient stimuli within our environment https://www.zotero.org/google-docs/?WIQqIp (Cole, 2015; Crouzet et al., 2010). Facial expressions and prosody are useful sources of information for communicating the status of interpersonal relationships, or signaling the presence of threatening agents, which may impose personal harm. Faces exhibiting aggression or fear and threatening prosody activate fear/threat circuitry, including the amygdala (de Gelr et al., 2004; Frühholz and Grandjean, 2013; Kret et al., 2011; Pichon et al., 2009). Depending upon the nature of threatening stimuli, quick detection within the environment and initializing defensive or avoidant behavioral strategies is evolutionarily advantageous. This initialization necessitates effective communication across the brain, which subserves cognitive, affective, and motoric processes. Not only does this require communication between singular (i.e., local) brain regions, but also between more widespread (i.e., global) intrinsic connectivity networks (ICNs). As intra-network communication reflects more local communication between singular regions or networks, inter-network communication reflects communication between regions or distinct networks on a global scale. As such, threat detection can be seen to engage processes of attention, sensory detection and categorization, affective saliency, motoric planning and motoric execution. Processes that engage multiple brain regions, but also components of attentional, sensory, limbic, saliency, motor and somatomotor ICNs. Consequently, threat detection involves an enormous amount of computations at both intra- and inter-network levels.

Human neuroimaging studies provide researchers with the ability to understand the neurobiological underpinnings of fear processing. A recent mega-analysis of 2,199 subjects who completed fear conditioning tasks found a large network of brain regions, including the dorsal anterior cingulate cortex (dACC), dorsolateral prefrontal cortex (dlPFC), somatosensory, anterior insula (aIns), amygdala, thalamus, brainstem, basal ganglia, premotor, and supplementary motor area (SMA; Radua et al., 2025), results similar to a previous meta-analysis on fear conditioning (Fullana et al., 2016). The SMA, precentral gyrus, dACC and aIns show an increased likelihood of activation in response to threatening stimuli oriented towards the observer (versus away) indicating the importance of the proximity of threat for the engagement of the ventral attention/salience and motor ICNs (Lima Portugal et al., 2020). Several investigations involving different temporal epochs (CS-US pairings) and threatening stimuli (i.e., affective imagery, shock) have indicated changes in activation and patterns of functional responses in the premotor cortex and SMA (Mirabella et al., 2024; Fullana et al., 2016; Radua et al., 2025; Wen et al., 2024). Activation of the premotor and SMA may indicate the generation of a preparatory motor response while attending to fear conditioned stimuli. Intuitively so, as threat induced motor preparation would likely be evolutionarily selected to ‘prime’ the motor system to escape or engage in threats. Globally, evidence across the previous studies employing stimuli evocative of potential harm or affectively averse imagery provides empirical evidence of attentional (dACC, dlPFC), salience and affective (aIns, amygdala), and somatomotor ICNs (somatosensory, premotor, SMA), indicating a hierarchical convergence of cognitive, affective and motoric processing to adequately respond to threat.

The aforementioned evidence provides support that, while numerous and at times apparently heterogeneous brain regions are involved in processing threat, a greater understanding of dynamic intra- and inter-network interaction of the brain can be gleaned from examining the network organization and characteristics of both local and global features. Therefore, mathematical procedures such as graph theory, a mainstay methodology of network neuroscience, provide an opportunity to interrogate the functional organization of network systems by transforming matrices of functional connectivity into a series of nodes (i.e., brain regions) and edges (i.e., measures of connectivity) (Sporns, 2013, 2018). By virtue, the use of graph theory to functionally partition the brain into distinct nodes, results in intra-network regions showing higher functional connectedness, as compared to inter-network regions, thus providing both local and global metrics of brain communication. Variation in a region’s local connectedness is thought to reflect network coherence, which is related to the graph measure of participation coefficient (Guimerà and Nunes Amaral, 2005). Network neuroscience further highlights the role of regions that bridge between local neighboring nodes (hubs; Weiller et al., 2025) to facilitate more efficient information flow, and is reflected by the graph metric betweenness centrality (Brandes, 2008). While participation coefficient and betweenness centrality are related to local communication, the organization of global connectivity can be characterized by assessing the interconnectedness of each region via the graph metric of global efficiency (Latora and Marchiori, 2001). Variations in global efficiency indicate the degree to which the brain, regardless of its network structure, are functionally communicating with each other. Similarly, spatial variability of interconnectedness is an intrinsic property of the brain, and is reflected by the graph metric of modularity (Newman and Girvan, 2004). Global networks with high modularity reflect well defined communities of interconnected regions.

Recent work, using graph theory, has identified a distributed network of hubs along medial and lateral surfaces of the brain, encompassing the default mode, ventral attention, and dorsal attention ICNs (Weiller et al., 2025). The amygdala has been characterized as a critical hub, particularly within the social, fear and emotion processing domains, and is thought to bolster information integration (Bickart et al., 2014; Fraenz et al., 2025; Gilpin et al., 2015; McFadyen, 2019), as well as other regions implicated as being critically integrative hubs including the aIns (Huang et al., 2021) and ACC (Tang et al., 2019). Functionally, both the aIns and ACC contribute to the integration of interoceptive and exteroceptive information for supporting affective and motoric responding to dynamic environments (Medford and Critchley, 2010). However, regions within the visual network exhibit increased betweenness centrality when participants were presented with threats within the visual sensory domain (Lithari et al., 2016). Individuals with social anxiety disorder, subsequently endorsing enhancing sensitivity to threatening social cues, show reductions in efficiency and participation coefficient within the parahippocampal gyrus, posterior cingulate cortex, dorsolateral prefrontal cortex, insula and the calcarine sulcus, which are identified as unique features when compared against healthy controls (Yang et al., 2019). During an electric shock threat task, global efficiency was found to be increased, in addition to decreased modularity reflecting increased inter-network connectivity during threat processing (Kinnison et al., 2012). In sum, these findings indicate that threat plays a role in promoting functional reorganization in support of appropriate behavioral responding.

Functional reorganization likely reflects changes in attentional prioritization toward potential harm. Within the *Integration Hypothesis* (Godwin et al., 2015; Sun et al., 2020), increased awareness is underpinned by increased inter-network functional connectivity which dedicates more neurocognitive resources to process and respond to salient features within the environment. Indeed, threat takes precedence over neutral and positive stimuli, and promotes attentional prioritization even when it is incongruent to an ongoing task (March et al., 2022; Van Steenbergen et al., 2011; Zsidó et al., 2023). Therefore, threatening stimuli serves as a highly salient feature promoting awareness. In such cases, research identifies the salience network (SN) as a critical factor for adjusting attentional prioritization and awareness. Effective connectivity analysis examining network hierarchy indicates that the (SN) resides atop a network hierarchy modulating the activity of the other networks (Zhou et al., 2018), highlighting its role in the integrative nature of network organization. Threat detection is cardinal to an organism and key regions of the SN have been identified as being active in threat processing (Fullana et al., 2016; Radua et al., 2025). Taken together, research shows the SN is an integral network for integration (inter-network connectivity) as well as highlighting the likely importance for inter-network connectivity in coordinating a response to salient information, like threat. Overall, the existing human neuroimaging literature using graph theory demonstrates its ability to distinguish fear processing, yet remains relatively underutilized as a tool to investigate differences in network functional organization.

In the current study, we sought to identify network organization when participants evaluated threatening, as compared to non-threatening stimuli, at both the local and global level. Initially, we employed univariate analyses to investigate the differences between connectivity during the processing of threatening vs. non-threatening stimuli using graph metrics of, (1) local connectivity: participation coefficient and betweenness centrality and, (2) global connectivity: modularity and global efficiency. This analysis provided the nodes that contribute to network dynamics. Subsequently, we used multivariate analysis to compare which nodes’ graph theory metric could classify threatening vs. non-threatening stimuli using machine learning via a support vector machine (SVM). We employed a whole brain analysis using SVM to supplement mass univariate analyses, which represents a large degree of the cognitive neuroscience literature (Poldrack et al., 2011), as it accounts for neighboring features. Thus, results from univariate analyses inform regional differences in threat processing while SVM provides additional context regarding the particular *uniqueness* of a region in comparison to its neighbors when evaluating threatening stimuli.

While the literature examining graph theory analysis of fear/threat tasks is scarce, our initial hypotheses are in accordance with the integration hypothesis (Godwin et al., 2015; Sun et al., 2020), as fear and threat perception likely involves integration across multiple neural networks (i.e., limbic, motor). Specifically, we hypothesized that regions within the somatomotor, ventral attention, and limbic ICNs would have increased participation coefficient in the fear as compared to neutral condition. This hypothesis reflects the expectation of decreased intra-network, but increased inter-network communication when responding to threatening stimuli that likely promote integration across multiple cognitive and behavioral domains. Furthermore, we hypothesized the betweenness centrality of regions contained in the default mode, ventral attention, and dorsal attention ICNs (Weiller et al., 2025), in addition to the amygdala (Bickart et al., 2014), would be significantly increased in the fear condition, as it is expected that inter-region connectivity would increase, thereby elevating the relative importance of integrative hubs. We further hypothesized that global efficiency would be greater during fear processing due to increased inter-network connectivity theoretically subserving threat detection and preparation (Kinnison et al., 2012). Lastly, we hypothesized that fear evaluation will decrease modularity, which reflects increased inter-network connectivity, thus reducing the intra-network connectivity of the brain (Kinnison et al., 2012). Regarding classification, we hypothesized that the most important features during classification would involve regions from attentional (dlPFC, dACC), affective (aIns, amygdala) and motor (premotor, SMA, somatomotor) ICNs.

## 2 Methods

The current paper compiled data from 4 separate studies from our research lab which investigated fear/threat detection/processing. Task A (*N* = 18): Threat Processing Task, Task B (*N* = 30): Threat Anticipation Task, Task C (*N* = 46): Social Threat Task, Task D (*N* = 30): Fear Acquisition, Extinction, and Reinstatement Task. Tasks A/B/D employed protocols to compare “fear” vs. “neutral” trials in differing paradigms to assess brain activation or connectivity during threat detection (Task A/C), threat ambiguity/certainty (Task B), or threat acquisition (Task D). In brief, experimental procedures are listed below (2.1–2.4), while expanded methods can be found for Task A, with minor variations for Tasks B/D (Naaz et al., 2019) and Task C (Knight et al., 2019).

### 2.1 Participants

Recruiting variables were identical across all datasets. Potential participants were identified through an advertisement placed on an online participant recruitment system, flyers posted around campus, and word-of-mouth. Participants were required to be at least 18 years old, right-handed, native English speakers, have normal or corrected-to-normal vision (e.g., glasses/contacts), and have no history of psychiatric and neurological disorders. Participants were excluded if they were left handed, take psychotropic medications which alter neurocognitive function, or endorsed a contraindication for MRI acquisition including: presence of ferrous metals in the body (e.g., pacemakers, cochlear implants, surgical clips, or metal fragments), serious medical conditions, pregnant, and claustrophobia. A total of 137 participants were recruited. One participant was excluded from dataset B due to a missing functional scan. In dataset C, 9 participants attrition from the dataset and 2 were missing either a T1 or functional file. Additionally, 1 participant had poor FreeSurfer reconstruction. In dataset D, 1 participant withdrew due to claustrophobia while undergoing scanning and 1 participant had poor FreeSurfer reconstruction. In sum, 124 participants were included in the final analysis (*M*_Age_ = 22.06, *SD*_Age_ = 4.79; *N_Female_* = 71). Participants were compensated for their time and were given an option to receive either course credit or $100 Amazon gift cards. All studies included in the present investigation were approved by the University of Louisville’s IRB (14.0811).

### 2.2 Stimuli

For Tasks A/B/D: Images of fearful and neutral faces (white and black, male and female faces) were acquired from the Chicago Face Database (Ma et al., 2015). Audio clips of aversive human screams (7 male, 7 female) were obtained from the Internet. Additionally, neutral noises of unintelligible conversational chatter in a coffee shop (neutral human sound) and nature sounds (running water and chirping birds) were found on the Internet. All audio clips were edited to 2000 ms in length and normalized for loudness using Audacity^1^. Red triangles and blue squares were used to cue the presentation of a fearful face with an aversive scream or a neutral face with a neutral sound, respectively. For Task C: Images of both male and female fearful (3/3) and neutral (2/2) human faces from the Eckman Facial Database (Ekman and Friesen, 1976). For all studies, during scanning, visual stimuli were displayed through E-Prime onto an Invivo Esys LCD TV monitor at the back of the scanner bore, which was viewed by participants through a mirror on the head-coil. Auditory stimuli were present binaurally through headphones at a predetermined constant level. Stimuli from each respective dataset were validated. For the Chicago Face dataset, a significant main effect of threat was found between neutral and fearful faces in task A (Naaz et al., 2019; *p* < 0.001), B (Knight, 2020; *p* < 0.001), and D (Cook, 2025*; p* < 0.001). For the Ekman dataset (Task C), a significant main effect of threat was found between neutral and fearful faces (*p* < 0.001 respectively).

### 2.3 Procedure

Task A: Each “fear” or “neutral” condition consisted of 24 trials, each trial contained a cue presented for 500 ms: a red triangle for the threat conditions and a blue square for neutral conditions. For Explicit threat or Neutral conditions, the cue was immediately followed by the simultaneous presentation of either a fearful face with a human scream, or a neutral face with conversational chatter, respectively. Stimuli (face and audio) were presented for a duration of 2000 ms, followed by an inter-trial interval (ITI) of 500 ms (trial duration: 4000 ms). All trails had an additional pseudorandom variable ITI (Jitter) to increase design efficiency for hemodynamic response estimation (0–14,000 ms). Task B: In total, the task included 20 ‘fear’ trials and 16 ‘neutral’ trials. Participants were provided with cue words (1,000 ms duration) to signal the impending condition. Fear trials were cued with the word ‘THREAT!’ or neutral trials with the word ‘SAFE!’. After cue presentation, a black screen briefly appeared for 500 s, always followed by the presentation of a fearful or neutral face accompanied by a human scream or conversational chatter, respectively (2000 ms) and an inter-trial interval (ITI) of 500 ms (total trial length = 4,000 ms). Task C: Images of both male and female fearful and neutral faces were presented for 4,000 ms in a pseudo-random order repeated 4 times. Task D: Each ‘fear’ or ‘neutral’ condition consisted of 24 trials. During a fear trial, a neutral face was presented for 1,000 ms, followed by the same face with a fearful expression and an accompanying gender-matched scream for 2000 ms. The neutral face was then shown again for 1,000 ms and, finally, a fixation cross for a pseudorandomized variable inter-trial interval. During neutral trials this same order was employed, however, the face never became fearful and the accompanying audio employed conversational chatter.

### 2.4 MRI acquisition

MRI data were collected from a 3 T Siemens Skyra scanner (20-channel head coil). Task A/B/C/D: Structural images were acquired from T1-weighted MPRAGE sequence: Voxel size = 0.8 mm^3^; TE = 2.26 ms; TR = 1700 ms; FoV = 204 mm. Task B/C/D: Functional BOLD images were collected using gradient-echo T2*-weighted echoplanar imaging (TR = 3,000 msec, TE = 30 msec, multiband accelerated Factor 2, FoV = 192 mm, 78 transverse slices with whole brain coverage, 1.5 mm3 voxels, flip angle = 90°). Slices were oriented obliquely along the AC–PC line. Task A: Functional images were acquired from T2*-weighted echo planar imaging: Voxel size = 3.2 mm^3^; TE = 28 ms; TR = 2000 ms; FoV = 204 mm. Foam padding was placed within the head coil, as needed, to minimize motion in all studies.

### 2.5 Neuroimaging processing

#### 2.5.1 Structural processing

Participant-specific regions of interest (ROIs) were produced using FreeSurfer (v7.4.1^2^) ran on a Linux research computing cluster running the RockyOS distribution (version 8). The technical details of these procedures are described in prior publications (Dale et al., 1999; Dale and Sereno, 1993; Fischl et al., 2001, 2004; Fischl et al., 1999a; Fischl et al., 1999b; Fischl and Dale, 2000; Han et al., 2006; Reuter et al., 2010, 2012; Ségonne et al., 2004).

Briefly, this processing includes removal of non-brain tissue using a hybrid watershed/surface deformation procedure (Ségonne et al., 2004), automated Talairach transformation, segmentation of the subcortical white matter and deep gray matter volumetric structures (Fischl et al., 2002; Fischl et al., 2004) intensity normalization (Sled et al., 1998), tessellation of the gray matter white matter boundary, automated topology correction (Fischl et al., 2001; Ségonne et al., 2007), and surface deformation following intensity gradients to optimally place the gray/white and gray/cerebrospinal fluid borders (Dale et al., 1999; Dale and Sereno, 1993; Fischl and Dale, 2000). Freesurfer morphometric procedures have been demonstrated to show good test–retest reliability across scanner manufacturers and across field strengths (Han et al., 2006; Reuter et al., 2012). We applied the 7-network, 400 parcel Schaefer-Yeo atlas (Schaefer et al., 2018) to generate a surface-based annotation of participant-specific regions of interest^3^. Participant-wise annotations were imported into CONN for functional connectivity analyses. Furthermore, we imported 12 subcortical regions (6 per hemisphere) taken from “aseg.mgz” to produce the default segmentation procedure. All subcortical regions were treated as their separate network. In sum, 412 ROIs across 8 defined networks were included in the present analyses.

#### 2.5.2 Functional connectivity processing

We used the default standardized preprocessing pipeline for surface-based connectivity within the CONN toolbox. Functional data were realigned using SPM’s realign and unwarp procedure (Andersson et al., 2001), which coregisters all functional volumes to the first acquired volume using a least squares and a 6 parameter (rigid body) transformation (Friston et al., 1995). Then, data were resampled using b-spline interpolation to correct for motion and magnetic susceptibility interactions. Outliers were identified using ART (Whitfield-Gabrieli et al., 2011) using conservative settings marking any framewise displacement above 0.9 mm or global BOLD signal changes exceeding 5 standard deviations as poor volumes (Nieto-Castanon, 2025; Power et al., 2014). While excluding all previously marked outliers, a reference functional image was constructed for each participant by averaging together all volumes. Lastly, functional and anatomical data were co-registered following a direct normalization procedure (Calhoun et al., 2017; Nieto-Castanon, 2025) using the unified segmentation and normalization algorithm provided by SPM (Ashburner, 2007; Ashburner and Friston, 2005) with the default IXI-548 tissue probability map template. Functional data were not smoothed due to its effect of artificially increasing functional connectivity between parcels, negatively impacting the validity of graph measures indexing centrality and connectedness (Alakörkkö et al., 2017). As such, we did not smooth functional data. Furthermore, we did not perform slice time correction to minimize data processing (Wu et al., 2011).

Data were denoised by regressing out non-neural sources of signal within the functional time series originating from motion and their first-order derivatives (12 factors), previously identified outliers, white-matter, and CSF using noise components identified through CompCor (Friston et al., 1996). Noise components were estimated by computing the average BOLD signal and largest principal components orthogonal to the BOLD average, motion parameters, and outlier scans within each subject’s eroded segmentation masks. Afterward, a high-pass filter of 0.008 (128 s) was applied to remove temporal signatures of slow-wave oscillations such as scanner drift, respirations, cardiac activity. We omitted a low-pass filter to minimize BOLD signal spill-over when evaluating condition contrasts.

### 2.6 Graph theory analyses

#### 2.6.1 Generating adjacency matrices and graph metrics

Graph analysis was completed using the Brain Connectivity Toolbox (Rubinov et al., 2009) in Matlab (R2024a, Natick, MA, US). Individual functional connectivity matrices were produced following the first-level analyses in CONN were imported into Matlab. Within network neuroscience, it is thought that preserving connection weight represents more reliable estimations of network dynamics (Hallquist and Hillary, 2018). As such, we created weighted adjacency matrices by applying varying levels of proportional thresholding between 1 and 50 percent (Servaas et al., 2013, 2015). Assessing networks across varying thresholds further minimizes the risk of statistical bias introduced through the arbitrary selection of cost without an *a priori* rationale. Negative edges present within the connectivity matrices were preserved by taking the absolute value before proportional thresholding.

Our study focused on network organization characteristics between fear and neutral conditions. As such, we selected local metrics of participation coefficient and betweenness centrality (see Table 1 for mathematical formulae). Participation coefficient assesses the relationship of nodal connectivity within a network consisting of community structure. Within network neuroscience, communities are represented by canonical networks each possessing a collection of unique nodes. Subsequently, each node has intra- and inter-network connectivity strength. Participation coefficient is a measure bounded between 0 and 1, with higher values representing more inter-network connectivity. Betweenness centrality is a measure of how often a given node rests within the shortest path between node pairs, inferring its importance in connecting neighbors to facilitate efficient information flow throughout the network. We used an unnormalized calculation of betweenness centrality, meaning that the possible values are between 0 and infinity. Functions used to identify both measures include a procedure which de-duplicated connectivity matrices to trim identical data. Measures of both participation coefficient and betweenness centrality were produced at each proportional threshold for both fear and neutral conditions. All calculations were collapsed within each condition to produce a *mean* participation coefficient and betweenness centrality for each participant to be evaluated statistically. A new matrix was constructed which possessed all averaged network measures and their respective task conditions for further analysis using support vector machine classification (SVM).

**Table 1:** Graph theory formula.

Measure	Formula
Participation coefficient	$PC_{i}=1-\sum_{s=1}^{N_{M}}{\left(\frac{k_{\text{is}}}{k_{i}}\right)}^{2}$
Global efficiency	$c_{B}(v)=\sum_{s\neq v\neq t}\frac{\sigma_{\mathrm{st}}(v)}{\sigma_{\mathrm{st}}}$
Modularity	$E_{\text{Global}}=\frac{1}{N(N-1)}\sum_{i\neq j}\frac{1}{d_{\mathrm{ij}}}$
Betweenness centrality	$Q=\frac{1}{2m}\sum (A_{\mathrm{ij}}-\frac{k_{i}k_{j}}{2m})\delta (c_{i},c_{j})$

Participation coefficient: $PC_{i}$ : Participation coefficient for node *i*. $k_{i}$ : Number of edges connected to node *i*. $k_{\text{is}}$ : Number of intranetwork edges connected to node *i*. $N_{M}$ : Total number of networks. $\sum_{s=1}^{N_{M}}$ : Summed across networks. Betweenness centrality: $c_{B}(v)$ : Betweenness centrality for node *v*. $\sum_{s\neq v\neq t}$ : Sum over all node pairs excluding node *v*. $\sigma_{\mathrm{st}}(v)$ : Count of shortest paths between nodes *s* and *t*, including node *v*. $\sigma_{\mathrm{st}}$ : Total count of all shortest paths between nodes *s* and *t*. Global efficiency: $E_{\text{Global}}$ : Global efficiency of system. N : Total number of nodes. N(N−1) : Number of distinct node pairs excluding self connections. $\sum_{i\neq j}$ : Sum across all unique pairs. $d_{\mathrm{ij}}$ : Shortest-path between nodes *i* and *j*. $\frac{1}{d_{\mathrm{ij}}}$ : Efficiency between nodes *i* and *j*. Modularity: Q : Modularity score of the system. $A_{\mathrm{ij}}$ : Presence of a connection between nodes *i* and *j*. $k_{i},k_{j}$ : Sum of weights of nodes *i* and *j*. m : Sum of all edge weights in system. $c_{i},c_{j}$ : Networks where nodes *i* and *j* belong. $\delta (c_{i},c_{j})$ : Binary flag whether node *i* and *j* belong to the same network.

We additionally analyzed global metrics of network organization to contextualize local metrics of participation coefficient and betweenness centrality. The interconnectedness of the whole brain is assessed through global efficiency, which takes the average of inverse distances between nodes. Modularity is characterized as a global measure reflecting the strength of community structure of an entire system (see Table 1 for mathematical formulae). Greater modularity reflects stronger definitions of communities, indicating increased compartmentalization of a system. Importantly, modularity within the Brain Connectivity Toolbox is assessed using the Newman’s spectral community detection procedure and does not consider the *a priori* construction of networks defined by the Schaefer-Yeo 400 parcel, 7 network atlas.

#### 2.6.2 Support vector machine (SVM) classification

We implemented a binary support vector machine classification (SVM) with a linear kernel using the “libsvm” package in MATLAB (Chang and Lin, 2011) to identify multivariate signatures distinguishing fear and neutral conditions. Binary SVM produces a high dimensional hyperplane identifying features which maximize the distance between classes. Data were stratified such that each fold contains approximately an equal number of participations from each dataset to minimize the risk for bias in subsequent classification accuracy. A linear kernel was selected to afford the opportunity to identify which features were most important for classification accuracy. Due to our sample size, we sought to maximize classification accuracy by implementing 4-fold nested cross validation rather than dividing the dataset into dedicated training and testing subgroups. In the outer-fold, the dataset was split into training and testing sets. The training set was used within the inner-fold to iteratively test *c* parameters using a stepsize of 1 to identify the best performing classifier defined by accuracy. The *c* parameter controls the penalization of misclassified labels with low values permitting more outliers to exist and higher values penalizing the presence of outliers. Afterward, the selected classifier was applied to the withheld testing set to generate the fold-specific classification accuracy. To increase the reliability of final classification accuracy, we completed a total of 1,000 permutations of 4-fold cross-validation for each graph theory metric. Measures of classification accuracy and feature weights were averaged across all 1,000 permutations.

### 2.7 Statistical analyses

#### 2.7.1 Univariate graph theory analyses

We investigated differences in network organization between fear and neutral conditions using a linear mixed-effects model (lmerTest version 3.1.3^4^; Kuznetsova et al., 2017) in R (version 4.5.2; Core Team R, 2024). We defined a fixed effect of condition (threat and neutral) and included a fixed covariate of age. A nested random effect modeled the interaction effect of participants within the 4 distinct datasets to account for inter-participant and inter-dataset variability present in the our models. Mass univariate hypothesis tests were conducted to assess participation coefficient and betweenness centrality independently. The package “emmeans” (version 2.0.0; Lenth and Piaskowski, 2026) was used to define an explicit contrast between fear and neutral conditions accounting for the confounding effect of age and nested random effects of dataset and participant. Results from the contrast test were treated as uncorrected findings and subsequently underwent FDR corrections to account for multiple comparisons within each hypothesis test (412 total). Two additional linear mixed-effects models were completed to compare global efficiency and modularity between fear and neutral conditions.

#### 2.7.2 SVM classification analyses

Stratified binary SVM classification was performed using local graph metrics (participation coefficient and betweenness centrality) and global (global efficiency and modularity) for classifying fear and neutral conditions. Classification performance was determined using accuracy and dice coefficients. Measure of accuracy was obtained through 1,000 permutations of nested-cross validated SVM to determine whether it is statistically better than random classification. New testing and training participants were identified at the start of each permutation. We compiled a null distribution of classification accuracy by randomly shuffling our dataset and repeating 1,000 permutations of nested cross-validation SVM to test for significance above chance. Subsequently, we calculated the probability of a randomly permuted classification accuracy being greater than the true classification accuracy. The significance threshold was set to *p* < 0.05. Particularly meaningful features for classification accuracy were identified by z-transforming support vector coefficients for standardization. Features weights exceeding a *z*-score of 1.96 (i.e., 95% confidence internal) were labeled as important nodes for classification accuracy.

## 3 Results

### 3.1 Univariate analyses

**Figure 1:**
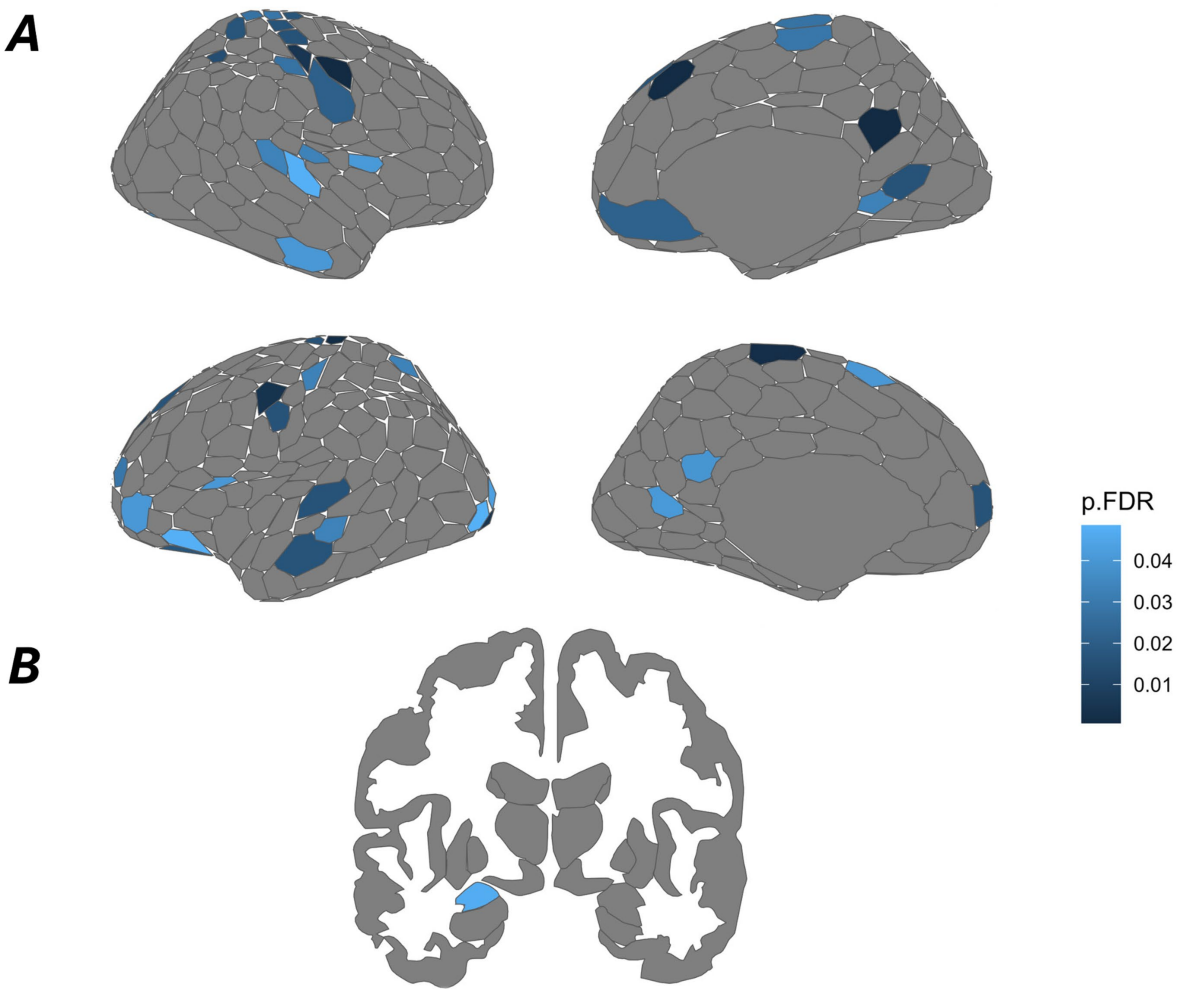
Mass univariate regressions for local network organization reveal distributed reductions in participation coefficient while evaluating threat. Each investigated ICN possesses at least one node which has a lower participation coefficient in the fear compared to the neutral condition. The two most represented ICNs are the somatomotor and default mode networks. All visualized regions are *p*_FDR_ < 0.05. Deeper shades of blue represent lower *p*-values. All presented *p*-values are below 0.05. **(A)** Cortical data is presented on the Schaefer-Yeo 7-Network 400 parcel atlas. **(B)** Subcortical data is presented on the default “aseg” atlas from FreeSurfer. The only significant subcortical structure was the left amygdala.

Local network organization was assessed using mass univariate analyses examining graph metrics of participation coefficient and betweenness centrality. We observed a diffuse pattern of significant effects of participation coefficient implicating at least one node of all 8 ICNS investigated in the present investigation. A total of 52 nodes had lower participation coefficients in the fear compared to the neutral condition. The plurality of nodes belong to the somatomotor ICN. No statistical difference in betweenness centrality between fear and neutral conditions were observed, *p*s > 0.26. Table 2 presents all significant findings (Figure 1).

**Table 2:** Results from univariate linear mixed model analyses for participation coefficient.

**Hemisphere**	**Region**	**Network**	***b***	***t*-value**	**p-FDR**
Left	Vis_12	Visual	0.022	4.086	0.005
	Vis_14	Visual	0.016	2.796	0.049
	Vis_19	Visual	0.019	2.858	0.043
	Vis_22	Visual	0.017	2.888	0.041
	SomMot_2	SomatoMotor	0.017	3.566	0.016
	SomMot_14	SomatoMotor	0.024	3.522	0.016
	SomMot_16	SomatoMotor	0.027	4.154	0.004
	SomMot_20	SomatoMotor	0.017	3.069	0.033
	SomMot_31	SomatoMotor	0.019	3.581	0.016
	SomMot_33	SomatoMotor	0.022	4.383	0.002
	SomMot_35	SomatoMotor	0.025	4.469	0.002
	SomMot_37	SomatoMotor	0.020	3.455	0.016
	Post_14	Dorsal Attention	0.012	3.022	0.033
	FrOperIns_7	Ventral Attention	0.010	2.902	0.040
	OFC_2	Limbic	0.013	3.384	0.017
	PFCl_1	Control	0.008	2.900	0.040
	PFCl_4	Control	0.010	3.131	0.029
	Temp_4	Default	0.014	3.480	0.016
	Temp_7	Default	0.016	3.026	0.033
	PFC_1	Default	0.012	2.805	0.048
	PFC_8	Default	0.015	3.508	0.016
	PFC_14	Default	0.019	3.947	0.006
	PFC_19	Default	0.015	3.431	0.017
	PFC_24	Default	0.013	2.911	0.040
	pCunPCC_3	Default	0.014	2.947	0.039
	Amygdala	Subcortical	0.010	2.788	0.049
Right	Vis_4	Visual	0.019	2.933	0.039
	Vis_14	Visual	0.022	3.061	0.033
	Vis_20	Visual	0.021	3.511	0.016
	SomMot_3	Somatomotor	0.014	2.805	0.048
	SomMot_6	Somatomotor	0.016	3.009	0.033
	SomMot_7	Somatomotor	0.014	3.038	0.033
	SomMot_16	Somatomotor	0.022	3.300	0.021
	SomMot_18	Somatomotor	0.028	4.915	0.001
	SomMot_19	Somatomotor	0.014	3.164	0.028
	SomMot_21	Somatomotor	0.025	4.020	0.005
	SomMot_25	Somatomotor	0.021	3.555	0.016
	SomMot_28	Somatomotor	0.013	3.413	0.017
	SomMot_29	Somatomotor	0.019	3.470	0.016
	SomMot_30	Somatomotor	0.018	3.118	0.029
	SomMot_33	Somatomotor	0.018	3.366	0.017
	SomMot_34	Somatomotor	0.017	3.118	0.029
	SomMot_35	Somatomotor	0.016	3.178	0.028
	SomMot_38	Somatomotor	0.020	3.142	0.028
	SomMot_40	Somatomotor	0.017	2.929	0.040
	Post_11	Dorsal attention	0.013	3.681	0.014
	FrOperIns_6	Ventral attention	0.013	2.882	0.041
	PFCmp_2	Control	0.018	4.699	0.001
	Temp_2	Default	0.013	2.918	0.040
	PFCdPFCm_1	Default	0.014	3.276	0.022
	PFCdPFCm_11	Default	0.016	3.403	0.017
	pCunPCC_4	Default	0.026	4.877	0.001

Results form univariate linear mixed model analyses for participation coefficient. All regions listed have significantly lower participation coefficient in the fear condition in contrast to the neutral condition (*p*s_FDR_ < 0.05). Lower fear participation coefficient indicates increased intra-network connectivity, suggesting enhanced intra-network coherence while evaluating threatening stimuli. All eight investigated networks are presented. The plurality (46.2%) of results are present in the somatomotor network.

**Figure 2:**
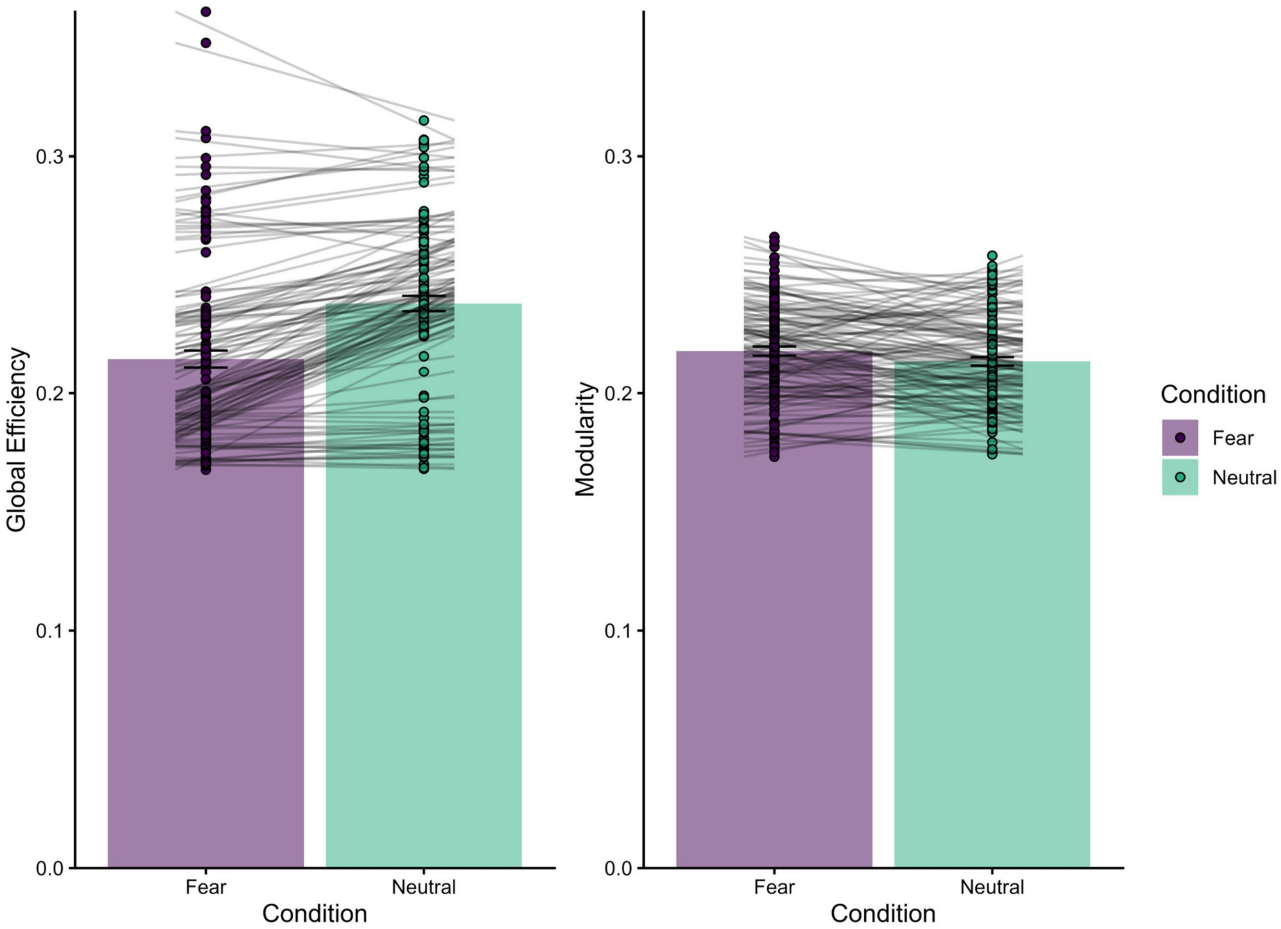
Univariate regressions for global network organization reveal differences in global efficiency and modularity while evaluating threat. Global efficiency (left panel) measures the interconnectedness of the whole brain (possible range 0–1). Global efficiency was significantly lower in the fear than neutral condition, suggesting less inter-network communication. Modularity (right panel) measures the strength of network definition within the brain with higher values indicating a more segregated structure (possible range 0–1). Modularity was significantly higher in the fear than neutral condition, suggesting greater intra-network communication. On average, the functional organization of individual networks were more defined within the fear than the neutral condition, indicating possible increased intra-network connectivity.

To assess global network organization we employed two additional linear mixed-effects models using graph measures of global efficiency and modularity between fear and neutral conditions (see Figure 2). Global efficiency was reduced in the fear compared to the neutral condition [*M*_diff_ = −0.023, *b* = 0.023, *SE* = 0.002, *t*(123) = 11.281, *p* < 0.001]. In contrast, global modularity was increased in the fear compared to the neutral condition [*M*_diff_ = 0.004, *b* = −0.044, *SE* = 0.002, *t*(123) = −2.613, *p* = 0.01].

### 3.2 Multivariate SVM classification

We next tested whether both the local and global network organization graph metrics could accurately predict fear from neutral conditions. Therefore, we conducted several 1,000-permutation nested cross-validation SVM to examine node-based classification.

#### 3.2.1 Local graph measures

**Figure 3:**
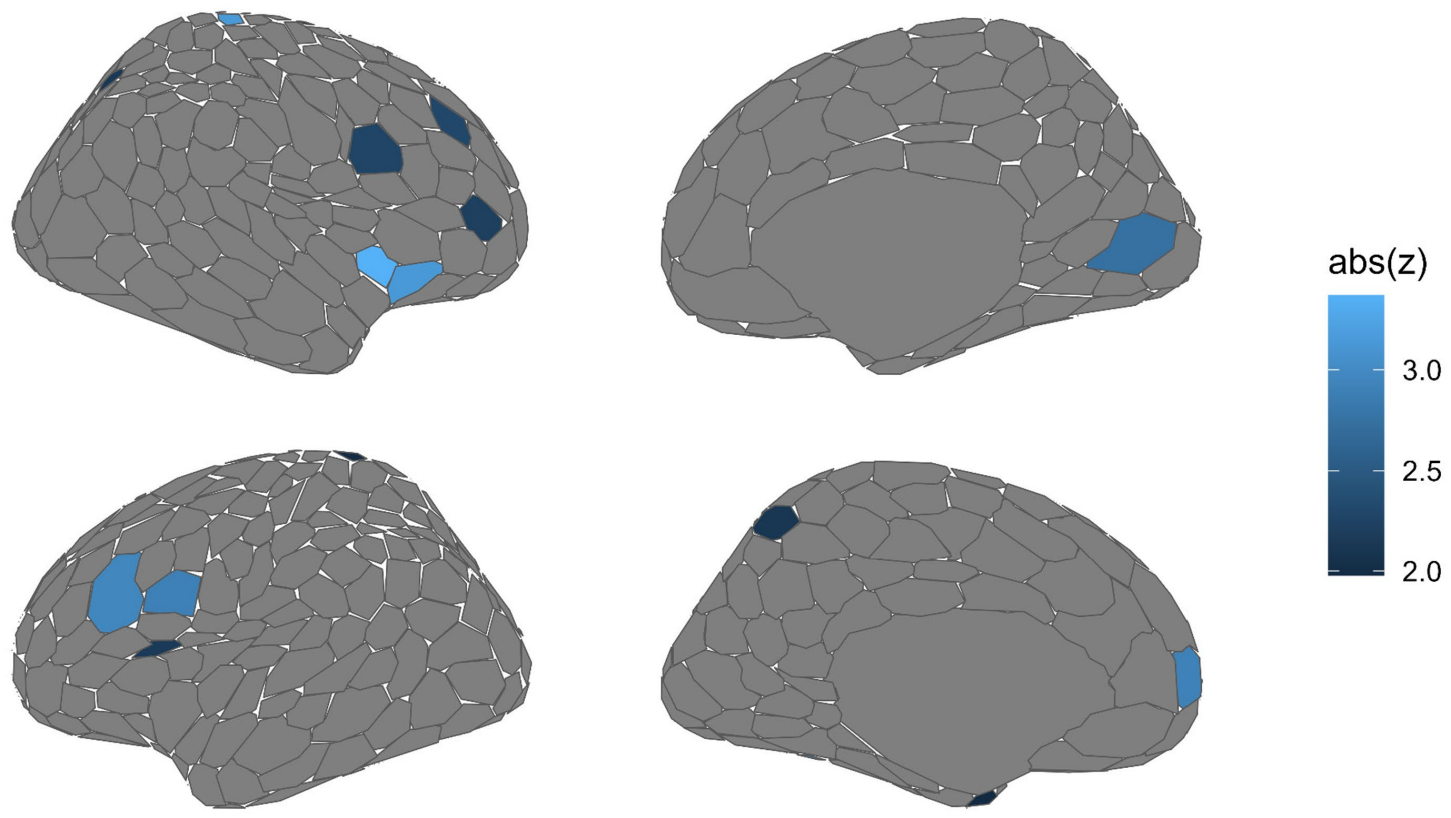
Brain regions important during classification of fear vs. neutral conditions based on local network organization graph measure of participation coefficient. Multivariate analyses using SVM reveal a distributed set of regions with participation coefficients that distinguish fear from neutral conditions. As with the univariate analyses, these regions are largely distributed across all networks, aside from the subcortex. The most important region for classification was the dorsal somatomotor region (RH SomMot_34, *z* = 3.975). Lighter shades of blue indicate higher *z* scores.

**Figure 4:**
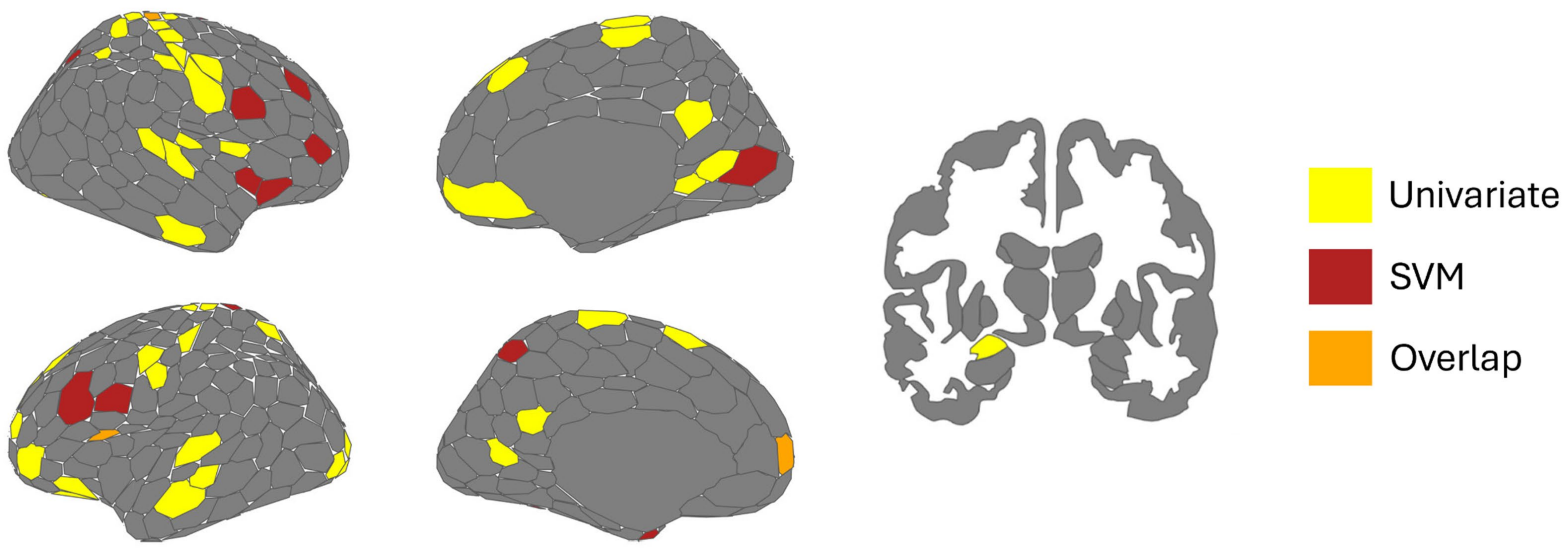
Regions identified across univariate and multivariate (SVM) analyses. All regions identified across univariate (yellow) and multivariate (red) analyses. Regions which overlap across both analyses are represented in orange. The dorsal somatomotor region (RH SomMot_34), fronto-operculum insula region (LH SalVentAttn_FrOperIns_7), and rostral medial prefrontal cortex (LH Default_PFC_8) are areas of converging evidence.

Analysis of the local network organization graph metric of participation coefficient exhibited low-moderate performance of 64.45%, which is significantly above chance (*p*_1000_ = 0.003), the obtained dice coefficient suggests moderate-to-high overlap between predicted and true labels (*Dice* = 0.629). A total of 16 regions exceeded a feature weight of *z* = 1.96, meeting our *a priori* definition of regions important for classifying fear from neutral conditions (see Table 3). Unlike the univariate analyses, important nodes were diffusely spread across all cortical ICNs. No node within the subcortical ICN was found to be important. The most important node was “RH SomMot_34” (*z* = 3.95). Figure 3 displays these results on the Schaefer-Yeo 7-Network 400 parcel atlas. A total of three regions identified from SVM were also present within the mass univariate analyses; “SomMot_34” (right hemisphere), “PFC_8” (left hemisphere), and “FrOperIns_7” (left hemisphere), belonging to the somatomotor, default mode, and ventral attention/salience ICNs, respectively (bolded in Table 3 and presented in Figure 4).

**Table 3:** Brain regions important during classification of fear vs. neutral conditions based on local network organization graph metric of participation coefficient.

Hemisphere	Region	Network	*z*-score
Left	Vis_3	Visual	2.793
	SomMot_36	Somatomotor	2.007
	PrCv_1	Dorsal attention	2.898
	**FrOperIns_7**	**Ventral attention**	**2.170**
	TempPole_2	Limbic	1.979
	PFCl_6	Control	2.962
	pCun_2	Control	2.121
	**PFC_8**	**Default**	**2.915**
Right	Vis_19	Visual	2.747
	**SomMot_34**	**Somatomotor**	**3.146**
	SomMot_36	Somatomotor	2.111
	Post_10	Dorsal attention	2.143
	PrCv_1	Dorsal attention	2.275
	FrOperIns_2	Ventral attention	3.368
	PFCv_1	Control	3.143
	PFCl_4	Control	2.220
	PFCl_11	Control	2.309

*Z*-Scored feature weights averaged across 1,000 permutations of nested-cross validation binary SVM which were greater than 1.96 are presented. Bolded regions represent overlap with mass univariate analyses.

Node-based classification accuracy for the local network organization graph metric of betweenness centrality between fear and neutral conditions was 45.88%, statistically indistinguishable from chance (*p*_1000_ = 0.681), the obtained dice coefficient suggests low overlap between predicted labels and true labels (*Dice* = 0.456).

#### 3.2.2 Global graph measures

**Figure 5:**
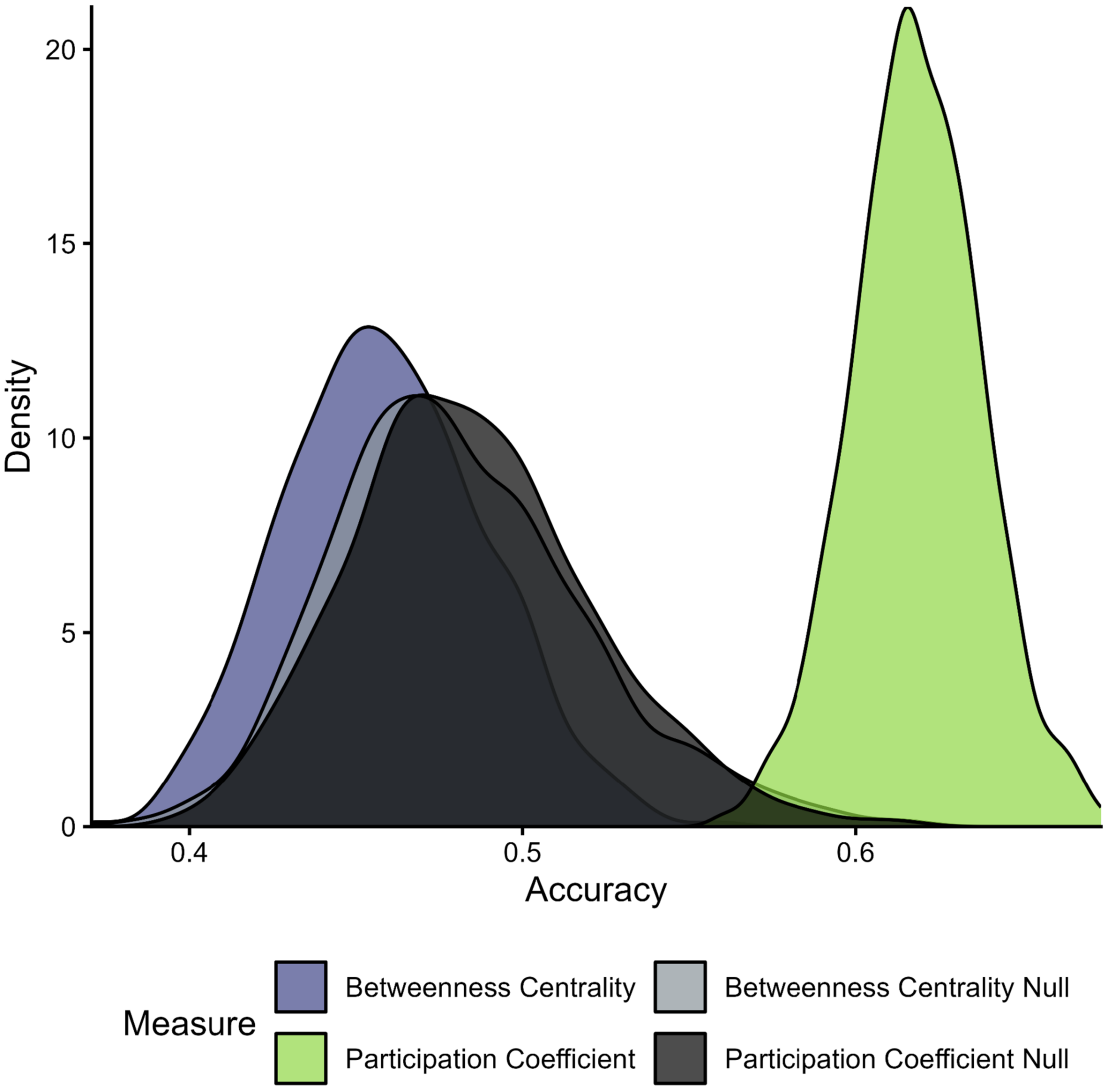
Accuracy distribution across all SVM permutations for local theory measures. Distribution of classification accuracy from 1,000 permutations for local graph theory metrics. The *Y*-axis depicts the number (or frequency) of permutations and the *X*-axis depicts the classification accuracy. The graph compares each distribution of permutations along classification accuracy. Null distributions for betweenness centrality (light gray) and participation coefficient (dark gray) are presented alongside the observed data for comparison. Global efficiency and modularity are not depicted due to the lack of meaningful inference caused by the uniform classification of “neutral” to all features across permutations.

Both global efficiency and modularity exhibited classification accuracy at 50%. Upon investigation, it was revealed that all features were predicted to represent the neutral condition. Overall, this represents poor distinguishability between fear and neutral conditions across global metrics identified in the current investigation. Consequently, we did not perform random permutation analyses. Dice coefficients for both global efficiency and modularity were 0.333 (Figure 5).

## 4 Discussion

This investigation compared functional network organization between conditions where participants evaluated fearful or neutral stimuli across four distinct internal datasets representing social fear/threat, as stimuli consisted of fearful faces and screams. Functional network organization was investigated using both global (modularity, global efficiency) and local (participation coefficient, betweenness centrality) graph metrics of connectivity. We observed decreased participation coefficient across a distributed set of regions, largely implicating the somatomotor and default mode ICNs. In addition, fearful faces induced reduced global efficiency and increased modularity. Machine learning analyses using binary SVM revealed parallel results, albeit implicating fewer regions overall. Mass univariate and binary SVM did not reveal differences in betweenness centrality between fear and neutral conditions. Overall, our results reveal distinct differences from our *a priori* hypotheses, in that both global (modularity, global efficiency) and local (participation coefficient) graph metrics revealed a more modular, segregated functional network organization, (i.e., enhanced intra-network vs. inter-network connectivity) for fear rather than neutral conditions. Our results provide evidence against the integration hypothesis (Godwin et al., 2015) in that the brain organized into more segregated networks supporting network specific functions rather than integration across networks. However, the specific regional and network constituents reflected partial support for our *a priori* hypotheses, in that both univariate and multivariate analyses revealed differences in attentional, salience, limbic, and somatomotor ICNs when comparing fear to neutral conditions. Below we expound on these findings and present implications with how fear/threat responding is conceptualized in human network neuroscience.

### 4.1 Univariate network organization

#### 4.1.1 Local graph metrics

Univariate analyses revealed 52 nodes distributed across all 8 investigated ICNs exhibiting decreased participation coefficient in response to fear stimuli, thus indicating increased intra-network connectivity of those nodes in contrast to the hypothesized increase in participation coefficient. The somatomotor ICN contributed 24 (46.2%) of all nodes with significantly decreased participant coefficient. Bilateral increases in intra-network connectivity in the somatomotor ICN as observed in the present investigation may reflect the preparation of a motor plan in response to fearful or threatening stimuli, similar to other research indicating this same perspective (Fullana et al., 2016; Mirabella et al., 2024; Radua et al., 2025; Wen et al., 2024). Similarly, recent reconceptualization of the somatomotor ICN suggests that this network is important for whole-body action planning which integrates goals, physiology and body movement consistent with this network comprising a ‘somato-cognitive action network’ (Gordon et al., 2023). Thus, the finding of a high preponderance of nodes within the somatomotor ICN suggests that fearful/threatening stimuli may induce preparatory cognitive action plans to avoid or engage with such salient features in the environment. Critically, such motoric preparation seemingly involves *increased* intra-network connectivity rather than becoming more affiliated with other networks contributing to fear processing. As such, fearful/threatening stimuli may induce partial isolation of networks to coordinate responses in a manner that prioritizes certain intra-network processing, as opposed to communication across wide-spread networks in an inter-network manner, perhaps consistent with the immediacy of required responses.

Similar to the somatomotor ICN, increased intra-network connectivity evidenced by significantly decreased participation coefficient, was seen in other ICNs, albeit more focally. Increased intra-network connectivity of regions consisting of the aIns and frontal operculum, inclusive to the saliency/ventral attention ICNs, potentially reflects their role in guiding attention toward salient features of the environment, supporting motoric preparation (Fullana et al., 2016; Hindi Attar et al., 2010; Picó-Pérez et al., 2019; Sklerov et al., 2019; Uddin et al., 2014). These regions have also been shown to signal the somatomotor and visual ICNs to respond more strongly to approaching threatening stimuli (deBorst and Gelder, 2022). Similarly, increased intra-network connectivity of the default mode ICN may be indicative of self-referential processing of the safety signal (Marstaller et al., 2016; Qi et al., 2024) while evaluating the outcome of threat/danger and its relation to the individual. Focal changes in functional connectivity within the dorsal attention and visual ICNs reflects attentional prioritization of goal-relevant information, like threatening stimuli presented in the present study (Lithari et al., 2016; Scolari et al., 2015; Vossel et al., 2014). Within the subcortical ICN, only the amygdala exhibited changes in intra-network connectivity between fear and neutral conditions. The amygdala is largely considered as a vital structure for processing socioemotional relevance (Bickart et al., 2014; Fraenz et al., 2025; McFadyen, 2019). While there is a consensus attributing the amygdala to detecting salient socioemotional features within the environment, recent work with the sub-nuclei of the amygdala provide evidence for their differential responding to different aspects of threat processing (Hrybouski et al., 2016; Mendoza-Franco et al., 2025; Wen et al., 2022). Future investigations including these sub-nuclei may better capture how the amygdala, as an ensemble, responds to threat. Decreased participation coefficient across nodes from all investigated ICNs provides evidence that these networks increase intra-network connectivity in response to threat. This increase in intra-network connectivity, particularly in the somatomotor and attention ICNs, reflects motoric preparation and processing of goal-directed, salient information, respectively, in response to threat. Despite the focality of the results, the increased intra-network connectivity of all ICNs supports the idea that these networks work in parallel to support affective processing and the preparation of action in response to threat rather than a single threat detection network.

In contrast to participation coefficient, we did not observe differences in betweenness centrality between fear and neutral conditions. Regions with high betweenness centrality act as a critical hub within a network for efficient information flow(Brandes, 2008). As betweenness centrality is important to classify local hubs important for inter-network communication, our results may be consistent with this null finding indicating increases in intra-network connectivity. In particular, sensitivity analyses reported within the Supplementary materials indicate that betweenness centrality effects were not generalizable across all 4 datasets in the present manuscript. We hypothesized that the amygdala would exhibit greater betweenness centrality when participants evaluated threat, because of previous findings implicating its role in modulating distributed networks (Bickart et al., 2014; Fraenz et al., 2025; Gilpin et al., 2015; McFadyen, 2019). However, the null findings of our present investigation may indicate that the amygdalar centrality is not as sensitive to extrinsic context. Indeed, recent evidence emphasizes that affective processing, which includes distinguishing features as threatening, does not require distinct neural states to manifest (Sachs et al., 2025). Instead, emerging research argues that cognition and behavior may not be supported by brain regions becoming active and subsequently facilitating a proscribed role within its relevant system; but rather, through a complex distributed system where neurobiological correlates of behavior are dependent on multiple factors, including extrinsic and intrinsic priors that may have preceded the transient threatening event (Misra and Pessoa, 2026). More research is clearly needed to establish the role of nodal hubs within the brain and the degree to which inter-network organization is structured during fear/threat processing.

#### 4.1.2 Global graph metrics

Coinciding with the local graph metric of participation coefficient, we observed decreased global efficiency, indicating reduced inter-network connectivity. While research has found increased global efficiency during emotion processing (Kinnison et al., 2012; Park et al., 2025). Other research indicates a disruption in global efficiency and increased modularity when fear is more imminent and dangerous (Haubrich and Nader, 2023). Consistent with decreases in global efficiency, and again coinciding with reductions in inter-network connectivity, we found that fear/threat processing is associated with increased modularity within the brain. Evidence links highly modularized brains to more adaptive emotion processing, inferred by symptom improvement among a cohort of individuals diagnosed with PTSD (Cisler et al., 2016). However, the relationship between adaptiveness and modularity may be dependent upon the distinct networks involved (Guassi Moreira et al., 2021), or under investigation. Indeed, representations of sensorimotor features appear to be dependent upon a two stage process requiring an initial pattern of intra-network coherence, then transitioning to diffuse internetwork connectivity during later stages of attentional processing (Veit et al., 2021).

Contrary to our *a priori* hypotheses, our analyses regarding both local and global graph metrics are consistent with a more modular and less globally efficient network organization promoting intra-, rather than inter-network connectivity. Our connectivity profile is similar to results from Sun et al. (2020) as our task-relevant threat stimuli was associated with increased intra-network connectivity rather than increased inter-network integration. Therefore, it may not be the case that inter-network connectivity, and perhaps decreased modular definition of networks subserving cognitive, affective, and somatomotor processing, is necessary to support fear/threat processing. Thus, substantial functional reorganization of integrative regions across networks may not be required to efficiently distribute information to support behavioral responding while under threat. While results from Veit et al. (2021) indicate internetwork connectivity as an expression of coordinated processing following initial detection, it is important to note that their findings report peaks in intranetwork activity during both early and late stages of attentional processing. As such, our findings may indicate increased processing or specialization of certain networks relevant to an immediate goal (e.g., fear/threat processing).

### 4.2 Multivariate network organization

The present study implemented machine learning via binary SVM to assess potential convergence between multivariate and univariate results across global and local metrics of functional organization, and whether these distinct features could classify the difference between fear and neutral conditions. Overlap between significant regions in the univariate with regions that differentiate between conditions strengthen the importance of these regions in the network characteristics unique to threat. SVM can be a flexible tool for revealing how structures distinguish over and above others for threat processing compared to neutral processing. Contrast this to univariate analyses which provide whether a region is different between conditions. This is important because the results from univariate analyses inform regional differences in threat processing while SVM provides additional context regarding the particular *uniqueness* of a region in comparison to its neighbors when evaluating threatening stimuli.

While SVM revealed distributed effects of increased intra-network connectivity, important regions which influenced the classification between fear and neutral conditions were focal and distributed across all ICNs beside the subcortical ICN. Notably, convergence between multivariate and univariate analyses were solely observed in the somatomotor (dorsal motor strip), default (medial prefrontal cortex), and ventral attention/salience (anterior frontal operculum) ICNs. As such, these results generally overlap with the perspective that regions supporting attentional processes, whether interoceptive or externally oriented, and motoric planning differ when actively evaluating threat (Gordon et al., 2023; Marstaller et al., 2016; Qi et al., 2024; Sklerov et al., 2019). In contrast to the univariate analysis, binary SVM classification of local metrics suggests that important regions for evaluating threat do not necessarily involve substantial network reorganization. SVM critically differs by forming a hyperplane most optimally distinguishing data classes (Chang and Lin, 2011; Cortes and Vapnik, 1995), subsequently considering shared influence between distinct regions. However, the degree of congruence between univariate and multivariate analyses may partially emerge from inherent differences in dimensionality (Cortes and Vapnik, 1995). For instance, each participant contributed 412 features per condition for local metrics. Dimensionality reduction may be useful for future investigations with sample sizes comparable to this study to reduce the presence of noise caused by multiple, low-influential features (Bi et al., 2003).

Multivariate analyses exhibited accuracy above chance for only participation coefficient, yet at a modest rate of 61.88%. Exceptional classification accuracy using MRI is possible, particularly in cases involving well delineated abnormalities such as tumors (De Benedictis et al., 2024). However, complex neurocognitive processes require decoding across several distributed systems supporting behavior, subsequently presenting a more difficult problem for machine learning classifiers. Null findings from our SVM analyses may indicate the adoption of a protracted psychological state marked by threat anticipation in parallel to active processing of presented stimuli (Adolphs, 2013). As a consequence of explicitly framing tasks under threat detection and the observation of threatening stimuli being rated higher in fear, it is possible that the theorized discrepancy in awareness between threatening and neutral stimuli were flattened. Therefore, our results may indicate an environment with repeatedly presented threats, individuals may have been anchored by the expectation of threat.

While less than exceptional, our obtained classification accuracies appear comparable to other similar works (Sacchet et al., 2015). Outcomes from the univariate analyses of global connectivity graph metrics reveal significant inter-participant variability (see Figure 2). As such, one possible explanation for our modest participation coefficient classification accuracy is the presence of heterogeneity within the sample. This may be a consequence of our nomothetic approach which presumes a universal pattern within a population. To address such possibility, alternative perspectives emphasizing the utility of identifying idiosyncratic signatures are gaining popularity within cognitive neuroscience (Finn et al., 2020), and are notable for future work. In particular, such work may be beneficial for improving classification accuracies for complex neurocognitive processes.

### 4.3 Limitations

We present some limitations, which can be viewed as directions for future research that may bolster the generalizability of our findings. In particular, there is inconsistency to the inclusion of negative functional connectivity weights within adjacency matrices (Hallquist and Hillary, 2018). While we advocate for their inclusion, many investigations do not include them (see Hallquist and Hillary, 2018 for a discussion of this topic). The lack of the SVM model to distinguish between conditions for all graph metrics but participation coefficient the classification accuracy of local measures like participation coefficient and betweenness centrality may be impacted by the sample size of the investigation, as we used 412 parcellations to divide the brain. Thus, overfitting becomes a concern in SVM analyses when the number of features exceeds the sample size (Cortes and Vapnik, 1995). Increasing sample size is beneficial to adequately power investigations with large numbers of parcellations in whole brain analyses. While we implemented nested cross-validation which identified the most optimal *c* parameter, which directly influences the degree of fit of support vectors separating classes, we cannot fully eliminate the possibility that low performance accuracy was partially influenced by noise induced by our feature-to-sample size ratio. SVM models are harder to intuitively interpret compared to penalized regression models, future investigations comparing the predictive power of such models should be investigated to better understand the validity of analysis methods for fMRI data. Additionally, understanding variation in emotion and/or stimuli can help disentangle whether our results are unique to threat processing, or are generalized to broader affective processes.

Furthermore, participants in ‘Task C’ included in the analyses had more passive viewing trials for fearful stimuli compared to neutral. This may influence the functional connectivity patterns of neutral passive viewing trials by inducing a negative psychological state. In addition, ‘Task C’ only used face stimuli compared to the other tasks which employed screams paired with face stimuli. This lack of audio stimuli could bias the contribution from this dataset as participants did not respond to a more rich sensory representation of the threat/fear stimuli. Lastly, ‘Task A’ functional MRI scans were obtained with larger voxel dimensions and a shorter TR compared to the other three tasks. This may further dilute the strength of our analysis as the signal from these scans may be more sensitive to activity relative to the others. That being said, we estimated the increase of power by including these participants, outweighed these limitations. Care was still taken to address this concern by nesting a random effect modeling the interaction effect of participants within the 4 distinct datasets to account for inter-participant and inter-dataset variability present in the our models.

## 5 Conclusion and future directions

In the present study, we compared functional network organization between fearful and neutral stimuli across four distinct internal datasets representing social fear/threat. Using both univariate graph metrics and multivariate binary stratified SVM classification, we found that all ICNs investigated showed decreased inter-network, and thus, increased intra-network connectivity and modularity of these networks in response to fear/threat stimuli. This provides evidence that these networks do not need to be strongly interconnected to adapt to evolving environments. Rather, the analyses suggest that increased coherence within each network subserves the function(s) of the respective network for adaptive responding to the environment. However, when taking into account the SVM results of sparse nodes classifying threat, the need for increased coherence may be network specific. This is exemplified by the few number of brain regions from the control network emerging from both univariate and multivariate analyses, which could indicate that the control network broadly does not rely on increased coherence for adaptive responding. Furthermore, threat response may induce a ‘heightened’ need for distinct modularity, as it can be viewed as a process closely tied to survival and thus, extremely important, as compared to other types of stimuli processing. Finally, perhaps the hierarchical nature of distinct networks may induce the amount of inter vs. intra-connectivity exhibited. In this view, networks more tied to concrete sensory processing requirements may exhibit increased intra-network connectivity, while networks upstream processing more abstract and holistic representations may exhibit increased inter-network connectivity. Future investigations using graph theory to understand network organization are warranted to tease apart the varying dynamics between intra- and inter-network connectivity, from a whole brain perspective In addition, investigations of threat processing using SVM should consider more varied stimuli and presentation formats to reduce expectations from forming and subsequently reducing distinguishing features within the signal. Other models, like penalized regressions, are worth exploring in future investigations for understanding what regions contribute predictive power to classify conditions.

## Data Availability

The raw data supporting the conclusions of this article will be made available by the authors, without undue reservation.

## Footnotes

1
https://www.audacityteam.org/

2
http://surfer.nmr.mgh.harvard.edu/

3 Parcellation files, including the Gaussian classifier required to apply the atlas to individual participants, can be found here: https://github.com/ThomasYeoLab/CBIG/tree/master/stable_projects/brain_parcellation/Schaefer2018_LocalGlobal

4
https://www.zotero.org/google-docs/?lJcA46

## References

[ref1] Adolphs R. (2013). The biology of fear. Curr. Biol. 23, R79–R93. doi: 10.1016/j.cub.2012.11.055, 23347946 PMC3595162

[ref2] Alakörkkö T. Saarimäki H. Glerean E. Saramäki J. Korhonen O. (2017). Effects of spatial smoothing on functional brain networks. Eur. J. Neurosci. 46, 2471–2480. doi: 10.1111/ejn.13717, 28922510 PMC5698731

[ref3] Andersson J. L. R. Hutton C. Ashburner J. Turner R. Friston K. (2001). Modeling geometric deformations in EPI time series. NeuroImage 13, 903–919. doi: 10.1006/nimg.2001.0746, 11304086

[ref4] Ashburner J. (2007). A fast diffeomorphic image registration algorithm. NeuroImage 38, 95–113. doi: 10.1016/j.neuroimage.2007.07.007, 17761438

[ref5] Ashburner J. Friston K. J. (2005). Unified segmentation. NeuroImage 26, 839–851. doi: 10.1016/j.neuroimage.2005.02.018, 15955494

[ref6] Bi J. Bennett K. P. Embrechts M. Breneman C. M. Song M. (2003). Dimensionality Reduction via Sparse Support Vector Machines, Journal of Machine Learning Research 3, 1229–1243.

[ref7] Bickart K. C. Dickerson B. C. Feldman Barrett L. (2014). The amygdala as a hub in brain networks that support social life. Neuropsychologia 63, 235–248. doi: 10.1016/j.neuropsychologia.2014.08.013, 25152530 PMC4981504

[ref8] Brandes U. (2008). On variants of shortest-path betweenness centrality and their generic computation. Soc. Networks 30, 136–145. doi: 10.1016/j.socnet.2007.11.001

[ref9] Calhoun V. D. Wager T. D. Krishnan A. Rosch K. S. Seymour K. E. Nebel M. B. . (2017). The impact of T1 versus EPI spatial normalization templates for fMRI data analyses. Hum. Brain Mapp. 38, 5331–5342. doi: 10.1002/hbm.23737, 28745021 PMC5565844

[ref10] Chang C.-C. Lin C.-J. (2011). LIBSVM: a library for support vector machines. ACM Trans. Intell. Syst. Technol. 2:27:1-27:27. doi: 10.1145/1961189.1961199

[ref11] Cisler J. M. Sigel B. A. Kramer T. L. Smitherman S. Vanderzee K. Pemberton J. . (2016). Modes of large-scale brain network organization during threat processing and posttraumatic stress disorder symptom reduction during TF-CBT among adolescent girls. PLoS One 11:e0159620. doi: 10.1371/journal.pone.0159620, 27505076 PMC4978452

[ref12] Cook O. (2025) Examining fear conditioning/extinction and the role of the amygdala in human neuroimaging. Dissertation, University of Louisville

[ref9001] Core Team R. (2024). R: a language and environment for statistical computing [computer software]. CRAN. Available online at: https://www.R-project.org/

[ref13] Cortes C. Vapnik V. (1995). Support-vector networks. Mach. Learn. 20, 273–297. doi: 10.1007/BF00994018

[ref14] Crouzet S. M. Kirchner H. Thorpe S. J. (2010). Fast saccades toward faces: face detection in just 100 ms. J. Vis. 10:16. doi: 10.1167/10.4.16, 20465335

[ref15] Dale A. M. Fischl B. Sereno M. I. (1999). Cortical surface-based analysis: I. Segmentation and surface reconstruction. NeuroImage 9, 179–194. doi: 10.1006/nimg.1998.0395 9931268

[ref16] Dale A. M. Sereno M. I. (1993). Improved localizadon of cortical activity by combining EEG and MEG with MRI cortical surface reconstruction: a linear approach. J. Cogn. Neurosci. 5, 162–176. doi: 10.1162/jocn.1993.5.2.162 23972151

[ref17] De Benedictis S. G. Gargano G. Settembre G. (2024). Enhanced MRI brain tumor detection and classification via topological data analysis and low-rank tensor decomposition. J. Comput. Math. Data Sci. 13:100103. doi: 10.1016/j.jcmds.2024.100103

[ref18] de Gelr B. Snyder J. Greve D. Gerard G. Hadjikhani N. (2004). Fear fosters flight: a mechanism for fear contagion when perceiving emotion expressed by a whole body. Proc. Natl. Acad. Sci. USA 101, 16701–16706. doi: 10.1073/pnas.0407042101, 15546983 PMC528902

[ref9002] Ekman P. Friesen W. V. (1976). Measuring facial movement. Environmental Psychology & Nonverbal Behavior 1, 56–75. doi: 10.1007/BF01115465

[ref19] Finn E. S. Glerean E. Khojandi A. Y. Nielson D. Molfese P. J. Handwerker D. A. . (2020). Idiosynchrony: from shared responses to individual differences during naturalistic neuroimaging. NeuroImage 215:116828. doi: 10.1016/j.neuroimage.2020.116828, 32276065 PMC7298885

[ref20] Fischl B. Dale A. M. (2000). Measuring the thickness of the human cerebral cortex from magnetic resonance images. Proc. Natl. Acad. Sci. 97, 11050–11055. doi: 10.1073/pnas.200033797, 10984517 PMC27146

[ref21] Fischl B. Liu A. Dale A. M. (2001). Automated manifold surgery: constructing geometrically accurate and topologically correct models of the human cerebral cortex. IEEE Trans. Med. Imaging 20, 70–80. doi: 10.1109/42.906426, 11293693

[ref9003] Fischl B. Salat D. H. Busa E. Albert M. Dieterich M. Haselgrove C. . (2002). Whole brain segmentation: automated labeling of neuroanatomical structures in the human brain. Neuron 33, 341–355. doi: 10.1016/s0896-6273(02)00569-x 11832223

[ref22] Fischl B. Sereno M. I. Dale A. M. (1999a). Cortical surface-based analysis. II: inflation, flattening, and a surface-based coordinate system. NeuroImage 9, 195–207. doi: 10.1006/nimg.1998.0396 9931269

[ref23] Fischl B. Sereno M. I. Tootell R. B. Dale A. M. (1999b). High-resolution intersubject averaging and a coordinate system for the cortical surface. Hum. Brain Mapp. 8, 272–284. doi: 10.1002/(sici)1097-0193(1999)8:4<272::aid-hbm10>3.0.co;2-4, 10619420 PMC6873338

[ref24] Fischl B. van der Kouwe A. Destrieux C. Halgren E. Ségonne F. Salat D. H. . (2004). Automatically parcellating the human cerebral cortex. Cereb. Cortex 14, 11–22. doi: 10.1093/cercor/bhg087 14654453

[ref25] Fraenz C. Metzen D. Merz C. J. Selpien H. Friedrich P. Ocklenburg S. . (2025). Fear learning sculpts functional brain connectivity at rest beyond the traditional fear network. Behav. Brain Res. 495:115764. doi: 10.1016/j.bbr.2025.115764, 40774647

[ref26] Friston K. J. Ashburner J. Frith C. D. Poline J. -B. Heather J. D. Frackowiak R. S. J. (1995). Spatial registration and normalization of images. Hum. Brain Mapp. 3, 165–189. doi: 10.1002/hbm.460030303

[ref27] Friston K. J. Williams S. Howard R. Frackowiak R. S. J. Turner R. (1996). Movement-related effects in fMRI time-series. Magn. Reson. Med. 35, 346–355. doi: 10.1002/mrm.1910350312, 8699946

[ref28] Frühholz S. Grandjean D. (2013). Amygdala subregions differentially respond and rapidly adapt to threatening voices. Cortex 49, 1394–1403. doi: 10.1016/j.cortex.2012.08.003, 22938844

[ref29] Fullana M. A. Harrison B. J. Soriano-Mas C. Vervliet B. Cardoner N. Àvila-Parcet A. . (2016). Neural signatures of human fear conditioning: an updated and extended meta-analysis of fMRI studies. Mol. Psychiatry 21, 500–508. doi: 10.1038/mp.2015.88, 26122585

[ref30] Gilpin N. W. Herman M. A. Roberto M. (2015). The central amygdala as an integrative hub for anxiety and alcohol use disorders. Biol. Psychiatry 77, 859–869. doi: 10.1016/j.biopsych.2014.09.008, 25433901 PMC4398579

[ref31] Godwin D. Barry R. L. Marois R. (2015). Breakdown of the brain’s functional network modularity with awareness. Proc. Natl. Acad. Sci. 112, 3799–3804. doi: 10.1073/pnas.1414466112, 25759440 PMC4378398

[ref32] Gordon E. M. Chauvin R. J. Van A. N. Rajesh A. Nielsen A. Newbold D. J. . (2023). A somato-cognitive action network alternates with effector regions in motor cortex. Nature 617, 351–359. doi: 10.1038/s41586-023-05964-2, 37076628 PMC10172144

[ref33] Guassi Moreira J. F. McLaughlin K. A. Silvers J. A. (2021). Characterizing the network architecture of emotion regulation neurodevelopment. Cereb. Cortex 31, 4140–4150. doi: 10.1093/cercor/bhab074, 33949645 PMC8521747

[ref34] Guimerà R. Nunes Amaral L. A. (2005). Functional cartography of complex metabolic networks. Nature 433, 895–900. doi: 10.1038/nature03288, 15729348 PMC2175124

[ref35] Hallquist M. N. Hillary F. G. (2018). Graph theory approaches to functional network organization in brain disorders: a critique for a brave new small-world. Network Neurosci. 3, 1–26. doi: 10.1162/netn_a_00054, 30793071 PMC6326733

[ref36] Han X. Jovicich J. Salat D. van der Kouwe A. Quinn B. Czanner S. . (2006). Reliability of MRI-derived measurements of human cerebral cortical thickness: the effects of field strength, scanner upgrade and manufacturer. NeuroImage 32, 180–194. doi: 10.1016/j.neuroimage.2006.02.051, 16651008

[ref9004] Haubrich J. Nader K. (2023). Network-level changes in the brain underlie fear memory strength. eLife 12:RP88172. doi: 10.7554/eLife.88172 38047914 PMC10695559

[ref9005] Hindi Attar C. Müller M. M. Andersen S. K. Büchel C. Rose M. (2010). Emotional processing in a salient motion context: integration of motion and emotion in both V5/hMT+ and the amygdala. J. Neurosci. 30, 5204–5210. doi: 10.1523/JNEUROSCI.5029-09.2010 20392942 PMC6632774

[ref9006] Hrybouski S. Aghamohammadi-Sereshki A. Madan C. R. Shafer A. T. Baron C. A. Seres P. . (2016). Amygdala subnuclei response and connectivity during emotional processing. NeuroImage 133, 98–110. doi: 10.1016/j.neuroimage.2016.02.056 26926791

[ref37] Huang Z. Tarnal V. Vlisides P. E. Janke E. L. McKinney A. M. Picton P. . (2021). Anterior insula regulates brain network transitions that gate conscious access. Cell Rep. 35:109081. doi: 10.1016/j.celrep.2021.109081, 33951427 PMC8157795

[ref38] Kinnison J. Padmala S. Choi J.-M. Pessoa L. (2012). Network analysis reveals increased integration during emotional and motivational processing. J. Neurosci. 32, 8361–8372. doi: 10.1523/JNEUROSCI.0821-12.2012, 22699916 PMC3400262

[ref39] Knight L. K.. (2020). Anxiety and how to control it: The functional role of the bed nucleus of the stria terminalis. Dissertation, University of Louisville

[ref9007] Knight L. K. Stoica T. Fogleman N. D. Depue B. E. (2019). Convergent neural correlates of empathy and anxiety during Socioemotional processing. Front. Hum. Neurosci. 13. doi: 10.3389/fnhum.2019.00094 PMC6438321 30949039

[ref40] Kret M. E. Pichon S. Grèzes J. de Gelr B. (2011). Similarities and differences in perceiving threat from dynamic faces and bodies. An fMRI study. NeuroImage 54, 1755–1762. doi: 10.1016/j.neuroimage.2010.08.012, 20723605

[ref41] Kuznetsova A. Brockhoff P. B. Christensen R. H. B. (2017). lmerTest package: tests in linear mixed effects models. J. Stat. Softw. 82, 1–26. doi: 10.18637/jss.v082.i13

[ref42] Latora V. Marchiori M. (2001). Efficient behavior of small-world networks. Phys. Rev. Lett. 87:198701. doi: 10.1103/PhysRevLett.87.198701, 11690461

[ref9008] Lenth R Piaskowski J (2026). Emmeans: estimated marginal means, aka least-squares means. R package version 2:2. Available online at: https://rvlenth.github.io/emmeans/

[ref9009] Lima Portugal L. C. S A. R. d. C. Junior O. F. Sanchez T. A. Mocaiber I. Volchan E. . (2020). Interactions between emotion and action in the brain. NeuroImage 214:116728. doi: 10.1016/j.neuroimage.2020.116728 32199954 PMC7485650

[ref43] Lithari C. Moratti S. Weisz N. (2016). Limbic areas are functionally decoupled and visual cortex takes a more central role during fear conditioning in humans. Sci. Rep. 6:29220. doi: 10.1038/srep29220, 27381479 PMC4933895

[ref1001] Ma D. S. Correll J. Wittenbrink B. (2015). The Chicago face database: a free stimulus set of faces and norming data. Behav. Res. Methods 47, 1122–1135. doi: 10.3758/s13428014-0532-5 25582810

[ref44] March D. S. Gaertner L. Olson M. A. (2022). On the automatic nature of threat: physiological and evaluative reactions to survival-threats outside conscious perception. Affect. Sci. 3, 135–144. doi: 10.1007/s42761-021-00090-6, 36046094 PMC9382976

[ref45] Marstaller L. Burianová H. Reutens D. C. (2016). Adaptive contextualization: a new role for the default mode network in affective learning. Hum. Brain Mapp. 38, 1082–1091. doi: 10.1002/hbm.23442, 27767246 PMC6867087

[ref46] McFadyen J. (2019). Investigating the subcortical route to the amygdala across species and in disordered fear responses. J. Experiment. Neurosci. 13:1179069519846445. doi: 10.1177/1179069519846445, 31068755 PMC6495431

[ref1002] Medford N. Critchley H. D. (2010). Conjoint activity of anterior insular and anterior cingulate cortex: awareness and response. Brain Struct. Funct. 214, 535–549. doi: 10.1007/s00429-010-0265-x 20512367 PMC2886906

[ref1003] Mendoza-Franco G. Tammilehto O. Jasinskaja-Lahti I. Aulbach M. B. Harjunen V. J. Peltola A. . (2025). The roles of amygdala subnuclei in processing of approaching in- and outgroup others in virtual space. Soc. Cogn. Affect. Neurosci. 20:nsaf119. doi: 10.1093/scan/nsaf119 41234053 PMC12673210

[ref47] Mirabella G. Tullo M. G. Sberna G. Galati G. (2024). Context matters: task relevance shapes neural responses to emotional facial expressions. Sci. Rep. 14:17859. doi: 10.1038/s41598-024-68803-y, 39090239 PMC11294555

[ref48] Misra J. Pessoa L. (2026). Human brain dynamics and spatiotemporal trajectories during threat processing. eLife 14:RP102539. doi: 10.7554/eLife.102539, 41609638 PMC12854673

[ref49] Naaz F. Knight L. K. Depue B. E. (2019). Explicit and ambiguous threat processing: functionally dissociable roles of the amygdala and bed nucleus of the stria terminalis. J. Cogn. Neurosci. 31, 543–559. doi: 10.1162/jocn_a_01369, 30605004

[ref50] Newman M. E. J. Girvan M. (2004). Finding and evaluating community structure in networks. Phys. Rev. E 69:026113. doi: 10.1103/PhysRevE.69.026113 14995526

[ref51] Nieto-Castanon A. (2025). “Preparing fMRI data for statistical analysis,” in fMRI Techniques and Protocols, ed. Filippi M. (New York, NY: Springer US), 163–191.

[ref52] Park J. S. Gollapudi K. Ke J. Nau M. Pappas I. Leong Y. C. (2025). Emotional arousal enhances narrative memories through functional integration of large-scale brain networks. Nat. Hum. Behav. 10, 370–383. doi: 10.1038/s41562-025-02315-1, 41083729

[ref53] Pichon S. de Gelder B. Grèzes J. (2009). Two different faces of threat. Comparing the neural systems for recognizing fear and anger in dynamic body expressions. NeuroImage 47, 1873–1883. doi: 10.1016/j.neuroimage.2009.03.084, 19371787

[ref1004] Picó-Pérez M. Alemany-Navarro M. Dunsmoor J. E. Radua J. Albajes-Eizagirre A. Vervliet B. . (2019). Common and distinct neural correlates of fear extinction and cognitive reappraisal: a meta-analysis of fMRI studies. Neurosci. Biobehav. Rev. 104, 102–115. doi: 10.1016/j.neubiorev.2019.06.029 31278951

[ref54] Poldrack R. A. Mumford J. A. Nichols T. E. (2011). Handbook of Functional MRI Data Analysis. Cambridge, UK: Cambridge University Press.

[ref55] Power J. D. Mitra A. Laumann T. O. Snyder A. Z. Schlaggar B. L. Petersen S. E. (2014). Methods to detect, characterize, and remove motion artifact in resting state fMRI. NeuroImage 84, 320–341. doi: 10.1016/j.neuroimage.2013.08.048, 23994314 PMC3849338

[ref56] Qi S. Cross L. Wise T. Sui X. O’Doherty J. Mobbs D. (2024). The role of the medial prefrontal cortex in spatial margin of safety calculations. J. Neurosci. 44:e1162222024. doi: 10.1523/JNEUROSCI.1162-22.2024, 38997158 PMC11340276

[ref57] Radua J. Savage H. S. Vilajosana E. Jamieson A. Abler B. Åhs F. . (2025). Neural correlates of human fear conditioning and sources of variability in 2199 individuals. Nat. Commun. 16:7869. doi: 10.1038/s41467-025-63078-x, 40849409 PMC12375119

[ref58] Reuter M. Rosas H. D. Fischl B. (2010). Highly accurate inverse consistent registration: a robust approach. NeuroImage 53, 1181–1196. doi: 10.1016/j.neuroimage.2010.07.020, 20637289 PMC2946852

[ref59] Reuter M. Schmansky N. J. Rosas H. D. Fischl B. (2012). Within-subject template estimation for unbiased longitudinal image analysis. NeuroImage 61, 1402–1418. doi: 10.1016/j.neuroimage.2012.02.084, 22430496 PMC3389460

[ref60] Rubinov M. Kötter R. Hagmann P. Sporns O. (2009). Brain connectivity toolbox: a collection of complex network measurements and brain connectivity datasets. NeuroImage 47:S169. doi: 10.1016/S1053-8119(09)71822-1

[ref61] Sacchet M. D. Prasad G. Foland-Ross L. C. Thompson P. M. Gotlib I. H. (2015). Support vector machine classification of major depressive disorder using diffusion-weighted neuroimaging and graph theory. Front. Psych. 6:21. doi: 10.3389/fpsyt.2015.00021, 25762941 PMC4332161

[ref62] Sachs M. E. Kozak M. S. Ochsner K. N. Baldassano C. (2025). Emotions in the brain are dynamic and contextually dependent: using music to measure affective transitions. eNeuro 12, ENEURO.0184–ENEU24.2025. doi: 10.1523/ENEURO.0184-24.2025, 40588361 PMC12243948

[ref63] Schaefer A. Kong R. Gordon E. M. Laumann T. O. Zuo X.-N. Holmes A. J. . (2018). Local-global parcellation of the human cerebral cortex from intrinsic functional connectivity MRI. Cereb. Cortex 28, 3095–3114. doi: 10.1093/cercor/bhx179, 28981612 PMC6095216

[ref64] Scolari M. Seidl-Rathkopf K. N. Kastner S. (2015). Functions of the human frontoparietal attention network: evidence from neuroimaging. Curr. Opin. Behav. Sci. Cognit. Control 1, 32–39. doi: 10.1016/j.cobeha.2014.08.003, 27398396 PMC4936532

[ref65] Ségonne F. Dale A. M. Busa E. Glessner M. Salat D. Hahn H. K. . (2004). A hybrid approach to the skull stripping problem in MRI. NeuroImage 22, 1060–1075. doi: 10.1016/j.neuroimage.2004.03.032, 15219578

[ref1006] Ségonne F. Pacheco J. Fischl B. (2007). Geometrically accurate topology-correction of cortical surfaces using nonseparating loops. IEEE Trans. Med. Imaging 26, 518–529. doi: 10.1109/TMI.2006.887364 17427739

[ref66] Servaas M. N. Geerligs L. Renken R. J. Marsman J.-B. C. Ormel J. Riese H. . (2015). Connectomics and neuroticism: an altered functional network organization. Neuropsychopharmacology 40, 296–304. doi: 10.1038/npp.2014.169, 25005250 PMC4443942

[ref67] Servaas M. N. van der Velde J. Costafreda S. G. Horton P. Ormel J. Riese H. . (2013). Neuroticism and the brain: a quantitative meta-analysis of neuroimaging studies investigating emotion processing. Neurosci. Biobehav. Rev. 37, 1518–1529. doi: 10.1016/j.neubiorev.2013.05.005, 23685122

[ref68] Sklerov M. Dayan E. Browner N. (2019). Functional neuroimaging of the central autonomic network: recent developments and clinical implications. Clin. Auton. Res. 29, 555–566. doi: 10.1007/s10286-018-0577-0, 30470943 PMC6858471

[ref69] Sled J. G. Zijdenbos A. P. Evans A. C. (1998). A nonparametric method for automatic correction of intensity nonuniformity in MRI data. IEEE Trans. Med. Imaging 17, 87–97. doi: 10.1109/42.668698, 9617910

[ref70] Sporns O. (2013). Structure and function of complex brain networks. Dialogues Clin. Neurosci. 15, 247–262. doi: 10.31887/dcns.2013.15.3/osporns 24174898 PMC3811098

[ref71] Sporns O. (2018). Graph theory methods: applications in brain networks. Dialogues Clin. Neurosci. 20, 111–121. doi: 10.31887/DCNS.2018.20.2/osporns, 30250388 PMC6136126

[ref72] Sun H. Yue Q. Sy J. L. Godwin D. Eaton H. P. Raghavan P. . (2020). Increase in internetwork functional connectivity in the human brain with attention capture. J. Neurophysiol. 124, 1885–1899. doi: 10.1152/jn.00693.2019, 33052763 PMC7814904

[ref73] Tang W. Jbabdi S. Zhu Z. Cottaar M. Grisot G. Lehman J. F. . (2019). A connectional hub in the rostral anterior cingulate cortex links areas of emotion and cognitive control. eLife 8:e43761. doi: 10.7554/eLife.43761, 31215864 PMC6624020

[ref1007] Uddin L. Q. Kinnison J. Pessoa L. Anderson M. L. (2014). Beyond the tripartite cognition–emotion–Interoception model of the human insular cortex. J. Cogn. Neurosci. 26, 16–27. doi: 10.1162/jocn_a_00462 23937691 PMC4074004

[ref74] Van Steenbergen H. Band G. P. H. Hommel B. (2011). Threat but not arousal narrows attention: evidence from pupil dilation and saccade control. Front. Psychol. 2:281. doi: 10.3389/fpsyg.2011.00281 22059081 PMC3204575

[ref75] Veit M. J. Kucyi A. Hu W. Zhang C. Zhao B. Guo Z. . (2021). Temporal order of signal propagation within and across intrinsic brain networks. Proc. Natl. Acad. Sci. 118:e2105031118. doi: 10.1073/pnas.2105031118, 34819365 PMC8640784

[ref76] Vossel S. Geng J. J. Fink G. R. (2014). Dorsal and ventral attention systems: distinct neural circuits but collaborative roles. Neuroscientist 20, 150–159. doi: 10.1177/1073858413494269, 23835449 PMC4107817

[ref77] Weiller C. Reisert M. Levan P. Hosp J. Coenen V. A. Rijntjes M. (2025). Hubs and interaction: the brain’s meta-loop. Cereb. Cortex 35:bhaf035. doi: 10.1093/cercor/bhaf035, 40077916 PMC11903256

[ref78] Wen Z. Pace-Schott E. F. Lazar S. W. Rosén J. Åhs F. Phelps E. A. . (2024). Distributed neural representations of conditioned threat in the human brain. Nat. Commun. 15:2231. doi: 10.1038/s41467-024-46508-0 38472184 PMC10933283

[ref1008] Wen Z. Raio C. M. Pace-Schott E. F. Lazar S. W. LeDoux J. E. Phelps E. A. . (2022). Temporally and anatomically specific contributions of the human amygdala to threat and safety learning. Proc. Natl. Acad. Sci. 119:e2204066119. doi: 10.1073/pnas.2204066119 35727981 PMC9245701

[ref79] Whitfield-Gabrieli S. Nieto-Castanon A. Ghosh S. (2011) Artifact Detection Tools (ART) (Version 7:11) [Computer Software]

[ref80] Wu C. W. Chen C.-L. Liu P.-Y. Chao Y.-P. Biswal B. B. Lin C.-P. (2011). Empirical evaluations of slice-timing, smoothing, and normalization effects in seed-based, resting-state functional magnetic resonance imaging analyses. Brain Connect. 1, 401–410. doi: 10.1089/brain.2011.0018, 22432454

[ref81] Yang X. Liu J. Meng Y. Xia M. Cui Z. Wu X. . (2019). Network analysis reveals disrupted functional brain circuitry in drug-naive social anxiety disorder. NeuroImage 190, 213–223. doi: 10.1016/j.neuroimage.2017.12.011, 29223742

[ref82] Zhou Y. Friston K. J. Zeidman P. Chen J. Li S. Razi A. (2018). The hierarchical Organization of the Default, dorsal attention and salience networks in adolescents and young adults. Cereb. Cortex 28, 726–737. doi: 10.1093/cercor/bhx307, 29161362 PMC5929108

[ref83] Zsidó A. N. Bali C. Kocsor F. Hout M. C. (2023). Task-irrelevant threatening information is harder to ignore than other valences. Emotion 23, 1606–1617. doi: 10.1037/emo0001189, 36355669

