# Supplementary material for: Investigating the functional neural network dynamics of cognitive-affective action planning during threat processing
Source: Front Psychol. 2026 Mar 27;17:1782939. doi: 10.3389/fpsyg.2026.1782939 (PMC13065709; doi:10.3389/fpsyg.2026.1782939)
Supplement: Supplementary file 1 [file Data_Sheet_1.pdf]

Supplementary material for "Investigating the Functional Neural Network Dynamics of Cognitive-Affective Action Planning during Threat Processing"

John Foley[1], Siraj J. Lyons[1], Olivia Cook[1,2], & Brendan E. Depue[1,3]*

[1] Department of Psychological & Brain Sciences, University of Louisville, Louisville KY, USA

[2] Surgical Theatre, Beechwood, OH, USA

[3] Department of Anatomical Sciences & Neurobiology, University of Louisville, Louisville KY, USA

* Corresponding Author:

Brendan E. Depue

brendan.depue@louisville.edu

University of Louisville

Supplementary Figure 1

Overlap between leave-one-study-out test

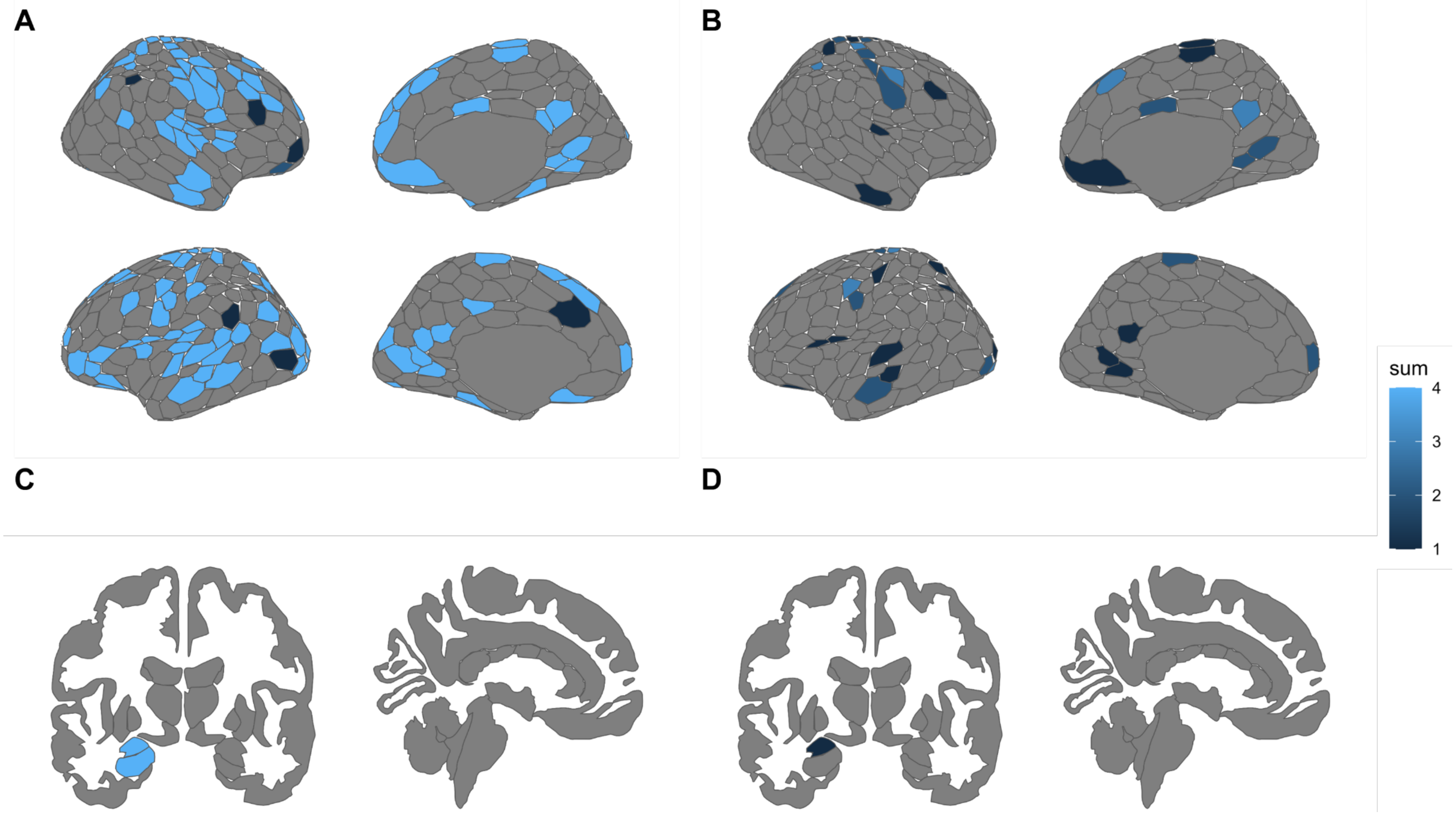

Cortical (A + B) and subcortical (C + D) results from leave-one-study-out analyses for participation coefficient (A + C) and betweenness centrality (B + D). Linear mixed effects models reported within the main text were kept unedited for these

supplemental analysis. We iteratively left out one study at a time to assess the stability of effects. The participation coefficient exhibited significant overlap across all studies with just a few regions exhibited study-dependent significance. In contrast, betweenness centrality exhibits general instability across studies. Furthermore, no significant regions were identified when “study C” was withheld from the analysis. Note that the nature of results in the supplementary analyses differ from the main text. As such, direct interpretation between the main text and these analyses are discouraged. Rather, these analyses solely represent the stability of our models across distinct datasets which vary in task stimuli and fMRI acquisition parameters.
